# Genes Influencing Phage Host Range in Staphylococcus aureus on a Species-Wide Scale

**DOI:** 10.1128/mSphere.01263-20

**Published:** 2021-01-13

**Authors:** Abraham G. Moller, Kyle Winston, Shiyu Ji, Junting Wang, Michelle N. Hargita Davis, Claudia R. Solís-Lemus, Timothy D. Read

**Affiliations:** a Microbiology and Molecular Genetics (MMG) Program, Graduate Division of Biological and Biomedical Sciences (GDBBS), Emory University, Atlanta, Georgia, USA; b Division of Infectious Diseases, Department of Medicine, Emory University, Atlanta, Georgia, USA; c Department of Epidemiology, Rollins School of Public Health (RSPH), Emory University, Atlanta, Georgia, USA; d Eugene Gangarosa Laboratory Research Fellowship, Emory College Online & Summer Programs, Emory College of Arts and Sciences, Atlanta, Georgia, USA; e Department of Statistics, University of Wisconsin-Madison, Madison, Wisconsin, USA; f Wisconsin Institute for Discovery, Department of Plant Pathology, University of Wisconsin-Madison, Madison, Wisconsin, USA; University of Rochester

**Keywords:** *Staphylococcus aureus*, bacteriophage lysis, bacteriophage therapy, bacteriophages, bioinformatics, computational biology, efficiency of plating, evolution, GWAS, phage host range, phage resistance, spot assay

## Abstract

Staphylococcus aureus is a human pathogen that causes serious diseases, ranging from skin infections to septic shock. Bacteriophages (phages) are both natural killers of S. aureus, offering therapeutic possibilities, and important vectors of horizontal gene transfer (HGT) in the species. Here, we used high-throughput approaches to understand the genetic basis of strain-to-strain variation in sensitivity to phages, which defines the host range. We screened 259 diverse S. aureus strains covering more than 40 sequence types for sensitivity to eight phages, which were representatives of the three phage classes that infect the species. The phages were variable in host range, each infecting between 73 and 257 strains. Using genome-wide association approaches, we identified putative loci that affect host range and validated their function using USA300 transposon knockouts. In addition to rediscovering known host range determinants, we found several previously unreported genes affecting bacterial growth during phage infection, including *trpA*, *phoR*, *isdB*, *sodM*, *fmtC*, and *relA*. We used the data from our host range matrix to develop predictive models that achieved between 40% and 95% accuracy. This work illustrates the complexity of the genetic basis for phage susceptibility in S. aureus but also shows that with more data, we may be able to understand much of the variation. With a knowledge of host range determination, we can rationally design phage therapy cocktails that target the broadest host range of S. aureus strains and address basic questions regarding phage-host interactions, such as the impact of phage on S. aureus evolution.

**IMPORTANCE** Staphylococcus aureus is a widespread, hospital- and community-acquired pathogen, many strains of which are antibiotic resistant. It causes diverse diseases, ranging from local to systemic infection, and affects both the skin and many internal organs, including the heart, lungs, bones, and brain. Its ubiquity, antibiotic resistance, and disease burden make new therapies urgent. One alternative therapy to antibiotics is phage therapy, in which viruses specific to infecting bacteria clear infection. In this work, we identified and validated S. aureus genes that influence phage host range—the number of strains a phage can infect and kill—by testing strains representative of the diversity of the S. aureus species for phage host range and associating the genome sequences of strains with host range. These findings together improved our understanding of how phage therapy works in the bacterium and improve prediction of phage therapy efficacy based on the predicted host range of the infecting strain.

## Abstract (precis)

Staphylococcus aureus is a widespread, hospital- and community-acquired pathogen, many strains of which are antibiotic resistant. It causes diverse diseases, ranging from local to systemic infection, and affects both the skin and many internal organs, including the heart, lungs, bones, and brain.

## INTRODUCTION

There is no licensed vaccine for Staphylococcus aureus, and many clinical strains are resistant to multiple antibiotics. For these reasons, alternative treatments such as bacteriophage therapy are being actively investigated (1, 2). Phage therapy has some advantages over using antibiotics. Phages show little or no human toxicity, and the high diversity of natural phages available to be isolated for treatment suggests that complete resistance would be hard to evolve (3, 4). However, there is no natural phage known to kill all S. aureus strains, and for that reason, phage cocktails (mixtures of phages with nonoverlapping host ranges) are necessary. Rational cocktail formulation requires comprehensive knowledge of the genetic factors that influence phage host range.

S. aureus phages and corresponding known host mechanisms regulating phage resistance and host range have previously been reviewed (1, 5, 6). Known S. aureus phages belong to the order *Caudovirales* (tailed phages) and are further divided into three morphological classes: the long, noncontractile-tailed *Siphoviridae*, the long, contractile-tailed *Myoviridae*, and the short, noncontractile-tailed *Podoviridae* (5). The *Siphoviridae* are temperate, while the *Myoviridae* and *Podoviridae* are virulent (5). The *Siphoviridae* bind either α-O-GlcNAc or β-O-GlcNAc attached at the four positions of wall teichoic acid (WTA) ribitol phosphate monomers, while the *Podoviridae* bind only β-O-GlcNAc-decorated WTA, and the *Myoviridae* bind either the WTA ribitol-phosphate backbone or β-O-GlcNAc-decorated WTA (1, 7, 8). S. aureus is known to produce polyribitol phosphate rather than polyglycerol phosphate WTA (9). WTA biosynthesis genes are conserved throughout the species, with the exception of the unusual sequence type ST395 (10), as are WTA glycosyltransferase genes *tarM* and *tarS*, but occasional *tarM* inactivation or absence provides *Podoviridae* susceptibility (11).

Currently identified resistance mechanisms in *Staphylococcus* species act at the adsorption, biosynthesis, and assembly stages of infection (1). Adsorption resistance mechanisms include receptor alteration, removal, or occlusion by large surface proteins or polysaccharides (capsule) (7, 11–16). Biosynthesis resistance mechanisms include halting the infection process through metabolic arrest (abortive infection) and adaptive (CRISPR) or innate (restriction-modification) immunity to phage infection through phage DNA degradation (17–21). Temperate phage and *S. aureus* pathogenicity islands (SaPIs) inserted in the genome may also offer barriers to *Siphoviridae*, through superinfection immunity and assembly interference, which occurs through SaPI parasitization of the packaging machinery of the infecting viruses (22–27).

While previous studies have identified numerous individual host resistance mechanisms in S. aureus, few have examined the importance of these mechanisms on a species-wide scale. In addition, although many S. aureus phages are reported to have wide host ranges (28–34), and even early studies suggested staphylococcal phage therapies to be highly effective (35), experiments conducted thus far have failed to explain the genetic bases of host range or resistance development in a species-wide manner. Only one previous study has associated genetic factors (gene families) with phage resistance by using a hypothesis-free method (36). This work used a two-step linear regression model to associate some 167 gene families, mostly of unknown function, with resistance assessed in 207 clinical methicillin-resistant S. aureus (MRSA) strains and 12 phage preparations. However, the study did not associate any other types of genetic changes with host range and examined only MRSA strains.

In this study, we associated multiple genetic factors—gene presence/absence, point mutations, and more complex polymorphisms—with S. aureus phage host range and resistance in a hypothesis-free, species-wide, and genome-wide manner. We used a novel high-throughput assay to determine resistance phenotypes of 259 strains challenged with eight S. aureus phages belonging to all three morphological categories (*Siphoviridae*, *Myoviridae*, and *Podoviridae*). We then used two bacterial genome-wide association study (GWAS) techniques to identify core genome single nucleotide polymorphisms (SNPs) and subsequences of length k (k-mers) significantly associated with each phenotype and used these significant features to develop predictive models for each phenotype. We also tested for associations between phenotypes and phylogeny, clonal complex (CC), and methicillin resistance (MRSA) and validated novel genes found to be associated with sensitivity or resistance in the GWAS through molecular genetics, thus complementing the hypothesis-free GWAS approach with hypothesis-driven experiments and demonstrating that GWAS-discovered determinants have causative effects on phage resistance.

## RESULTS

### Development of a novel high-throughput host range assay.

**FIG 1:**
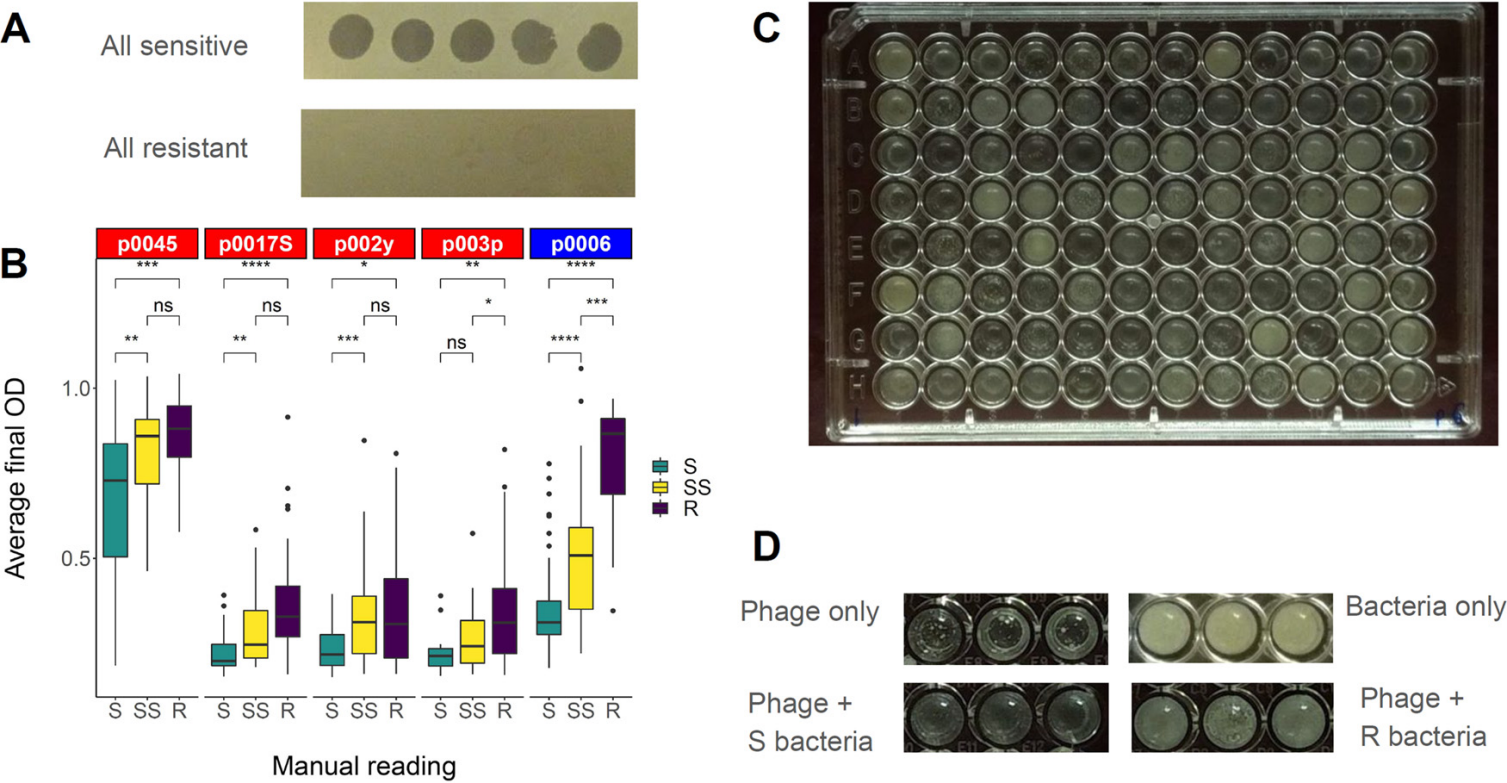
Development of the high-throughput phage host range assay. (A) Examples of fully sensitive (NRS149) and fully resistant (NRS148) spot assay phenotypes for five test phages (p0045, p0006, p0017S, p002y, and p003p). (B) Calibration of the high-throughput assay against qualitative spot assay phenotypes (S, sensitive, complete clearing; SS, semisensitive, cloudy clearing; R, resistant, no clearing) determined with the spot assay for 108 NARSA strains and the five phages listed for panel A. *Siphoviridae* are listed in red, and *Myoviridae* are listed in blue. Data represent the distribution of average high-throughput assay measurements for strains evaluated as S, SS, or R in corresponding spot assays. Wilcoxon signed-rank test significance values for each possible comparison are listed at the top of the corresponding box plots (ns, not significant; *, *P* = 0.01 to 0.05; **, *P* = 0.001 to 0.01; ***, *P* = 0.0001 to 0.001; ****, *P* = 0 to 0.0001). (C) Example of high-throughput assay results from one 96-well plate containing overnight cultures of 96 NARSA strains coincubated with phage p0006. (D) Example of high-throughput assay phenotypes for a sensitive S. aureus strain, a resistant strain, bacteria without phage, and phage without bacteria.

In order to evaluate host range for a large number of S. aureus strains in a quantitative manner, we developed a high-throughput host range assay (Fig. 1), described in Materials and Methods. This assay measures the extent to which phages cause retardation of growth compared to a control. Before using data from the high-throughput assay for further analysis, we calibrated it against the traditional spot assay (Fig. 1A), which measures whether phages cause lysis in a lawn of bacterial cells. We compared spot assay results (sensitive [S], semisensitive [SS], or resistant [R]) for 108 strains and five phages to the average final soft agar turbidity (optical density at 600 nm [OD_600_]) of the strains in the high-throughput assay (Fig. 1B). For all phages tested, turbidity was significantly higher (*P* < 0.05, Wilcoxon signed-rank test) for spot-resistant strains than for spot-sensitive strains. For all phages tested but p003p, the turbidity was significantly higher for spot-semisensitive strains than for spot-sensitive strains. However, for only phages p0006 and p003p were turbidities significantly higher for spot-resistant strains than for spot-semisensitive strains. Thus, for all phages but p003p, it was possible to tell spot-sensitive from spot-semisensitive strains by the high-throughput assay, but only for phages p0006 and p003p was it possible to tell spot-semisensitive from spot-resistant strains by the new assay. Overall, these results showed strong agreement between the lysis-based spot assay and the high-throughput growth-based assay for differentiating between sensitive and resistant/semisensitive phenotypes.

### Host range is associated with clonal complex but not methicillin resistance.

We evaluated the host range of eight phages belonging to the *Siphoviridae*, *Myoviridae*, and *Podoviridae*. *Siphoviridae* (p0045, p0017S, p002y, p003p, and p0040), *Myoviridae* (p0006 and pyo), and *Podoviridae* (p0017) were most closely related to others of the same class but were not related at all to those of other classes (see Table S1 at https://figshare.com/articles/dataset/Supplemental_Table_S1/13355909). Among the *Siphoviridae*, p003p was the most divergent from the others (between 97.75 and 97.83% similar to the others). On the host side, host range was determined for a set of 259 S. aureus strains representing 47 already defined sequence types (STs) and 17 already defined clonal complexes (CCs) against eight phages (253 strains with sequence data are included in Table S2 at https://figshare.com/articles/dataset/Supplemental_Table_S2/13355933). The most common STs were 5 (25.69%), 8 (13.04%), 30 (6.72%), 105 (4.35%), and 121 (3.16%), while the most common CCs were 5 (37.15%), 8 (23.32%), 30 (12.25%), 121 (5.14%), and 1 (4.74%), respectively. The most common strain isolation years were 2005 (31.92%), 2012 (14.08%), 2002 (12.68%), 2017 (7.51%), and 2018 (7.04%), while the most common isolation locations were the United States (61.26%), France (19.76%), the United Kingdom (11.46%), and Japan (1.19%). Strain isolation years ranged from 1935 to 2018.

**FIG 2:**
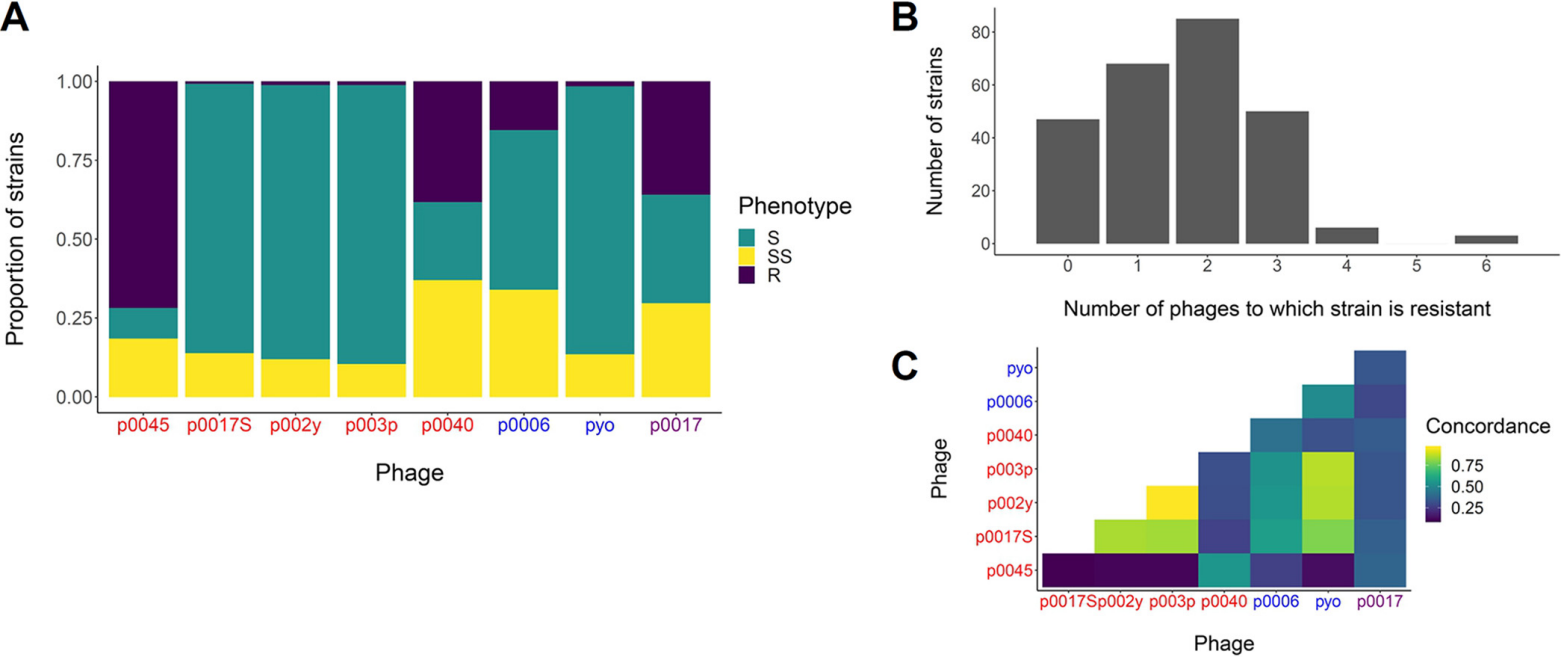
Host range distribution, concordance, and multiple phage resistance. (A) Number of strains that fall into host range categories for each phage. Sensitive (S) corresponds to an OD_600_ of 0.1 to 0.4, semisensitive (SS) corresponds to an OD_600_ of 0.4 to 0.7, and resistant (R) corresponds to an OD_600_ of 0.7 or higher. (B) Histogram of number of phages to which strains are resistant, by the previous definition. (C) Concordance matrix of the host ranges of the tested phages. Concordance is defined as the number of strains with identical phenotypes between two phages. *Siphoviridae* are listed in red, *Myoviridae* in blue, and *Podoviridae* in purple.

Phages p0045 and p0040, i.e., the two temperate phages, and p0017, the sole tested podovirus, had the highest proportions of resistant strains (71.8, 38.2, and 35.9%, respectively) among those tested (Fig. 2A and Table 1). The average and median final turbidities among tested strains were likewise highest for these phages (average/median, 0.80/0.88, 0.61/0.60, and 0.56/0.54, for p0045, p0040, and p0017, respectively). On the other hand, phages p0017S, p002y, p003p, and pyo, all virulent *Siphoviridae* or *Myoviridae*, had the lowest proportions of resistant strains (0.8, 1.2, 1.2, and 1.5%, respectively) and average/median final turbidities (0.31/0.27, 0.27/0.22, 0.32/0.31, and 0.26/0.21, respectively). Phage p0006 had an intermediate proportion of resistant strains (15.4%) and average/median final turbidity (0.49/0.44). Strains were resistant to between zero and six phages (Fig. 2B), with a median of two. The strains NRS148, NRS209, and NRS255 were resistant to six phages, the most among any strains. Phage host ranges were most similar (concordant, defined by number of strains with identical phenotypes between two phages) between phages p0017S, p002y, p003p, and pyo but least similar between phage p0045 and the previous set of four phages (Fig. 2C).

**TABLE 1:** Summary statistics of phage host range phenotypes^*a*^

Phenotype	No. (%) of strains with indicated phenotype to phage:							
	p0045	p0006	p0017	p0017S	p002y	p003p	p0040	Pyo
Sensitive	25 (9.7)	131 (50.6)	89 (34.4)	221 (85.3)	225 (86.9)	229 (88.4)	64 (24.7)	220 (84.9)
Semisensitive	48 (18.5)	88 (34.0)	77 (29.7)	36 (13.9)	31 (12.0)	27 (10.4)	96 (37.1)	35 (13.5)
Resistant	186 (71.8)	40 (15.4)	93 (35.9)	2 (0.7)	3 (1.2)	3 (1.2)	99 (38.2)	4 (1.5)
								
Mean	0.80	0.49	0.56	0.31	0.27	0.32	0.61	0.26
SD	0.24	0.20	0.27	0.12	0.12	0.12	0.23	0.14
Median	0.88	0.44	0.54	0.27	0.22	0.31	0.60	0.21

a For each phage, the number of strains falling into each phenotype category were counted. These phenotypes were determined for each phage using the high-throughput assay. The numbers of sensitive (OD_600_, 0.1 to 0.4), semisensitive (0.4 to 0.7), and resistant (0.7 and higher) strains and percentages are listed first, followed by the mean, standard deviation (SD), and median quantitative phenotypes for all tested strains. Statistics summarize at least six biological replicates for each phage.

**FIG 3:**
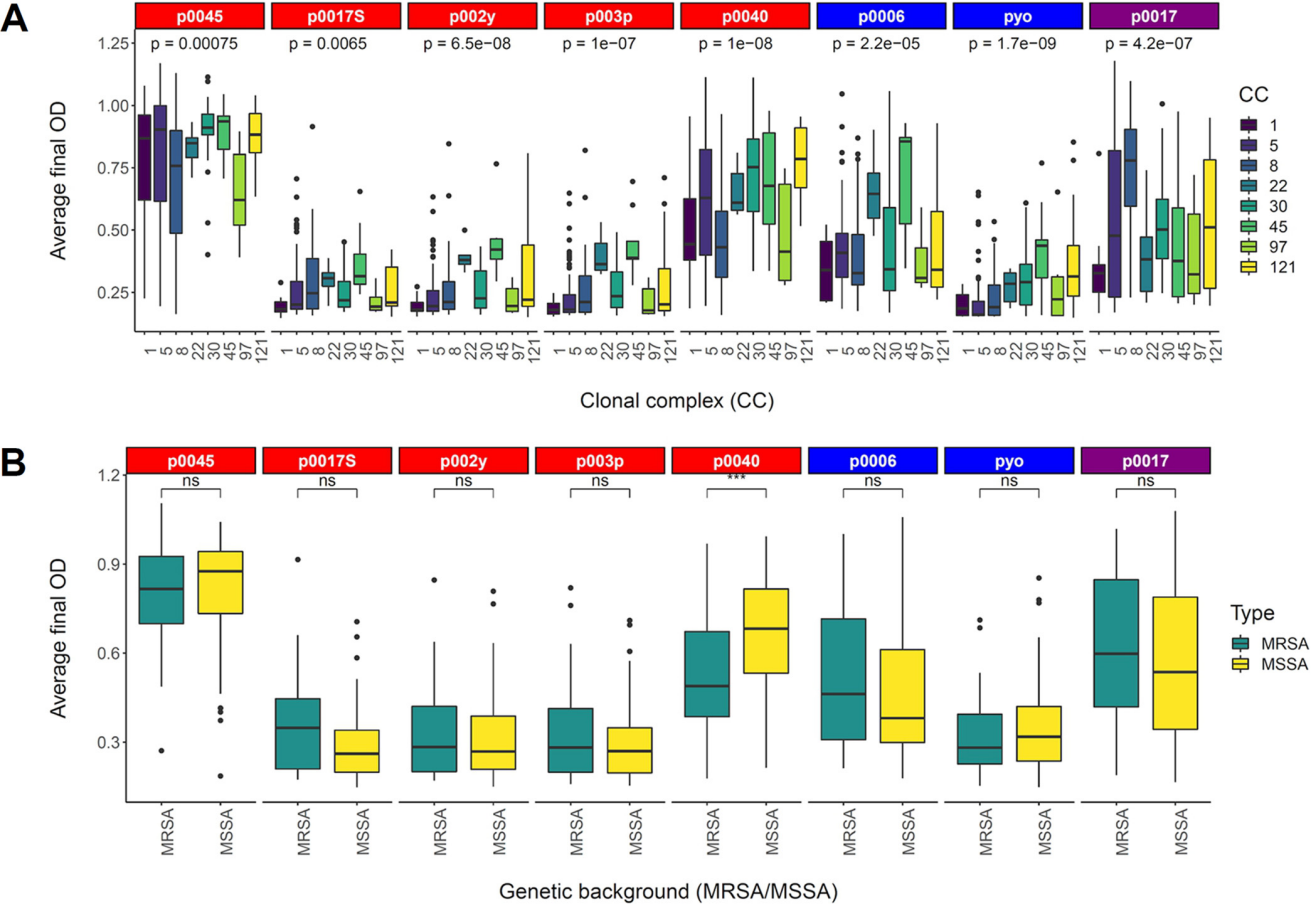
Phage resistance is related to clonal complex (CC) but not MRSA genetic background. Data represent the distribution of average high-throughput assay measurements for strains belonging to each presented CC (all 259 strains) (A) or MRSA/MSSA (126 NARSA strains) (B) genetic background. One-way ANOVA significance values for overall differences among CCs presented and Wilcoxon signed-rank test significance values for MRSA/MSSA differences are indicated at the top of the corresponding box plots (ns, not significant; *, *P* = 0.01 to 0.05; **, *P* = 0.001 to 0.01; ***, *P* = 0.0001 to 0.001; ****, *P* = 0 to 0.0001). *Siphoviridae* are listed in red, *Myoviridae* in blue, and *Podoviridae* in purple.

We also examined whether there were significant associations between clonal complex (CC) or methicillin-resistant S. aureus (MRSA) genetic background and each phage host range phenotype (Fig. 3). We hypothesized that CC would correlate with host range, given that type I restriction-modification specificity is strongly associated with CC (37, 38), restricting the infection of a strain by phage propagated in a strain of a different CC. We hypothesized that MRSA genetic background may also affect host range, given that the phage receptor WTA is required for methicillin resistance (39) but MRSA strains can tolerate more defects in WTA biosynthesis than methicillin-susceptible S. aureus (MSSA) strains (40). However, MRSA/MSSA phenotypic differences were only significant for phage p0040 (*P* < 0.001, Wilcoxon signed-rank test) (Fig. 3B). There were significant differences in phage resistance between individual CCs for all phages (*P* < 0.05, Tukey’s honestly significant differences based on one-way analysis of variance [ANOVA]) (see Table S4 at https://figshare.com/articles/dataset/Supplemental_Table_S4/13355942) and significant overall differences among all CCs (one-way ANOVA) for all phages (*P* < 0.05). Overall, these results indicate that MRSA genetic background for the most part is not associated with the host range of these phages, while CC overall affects the host ranges of all tested phages.

**FIG 4:**
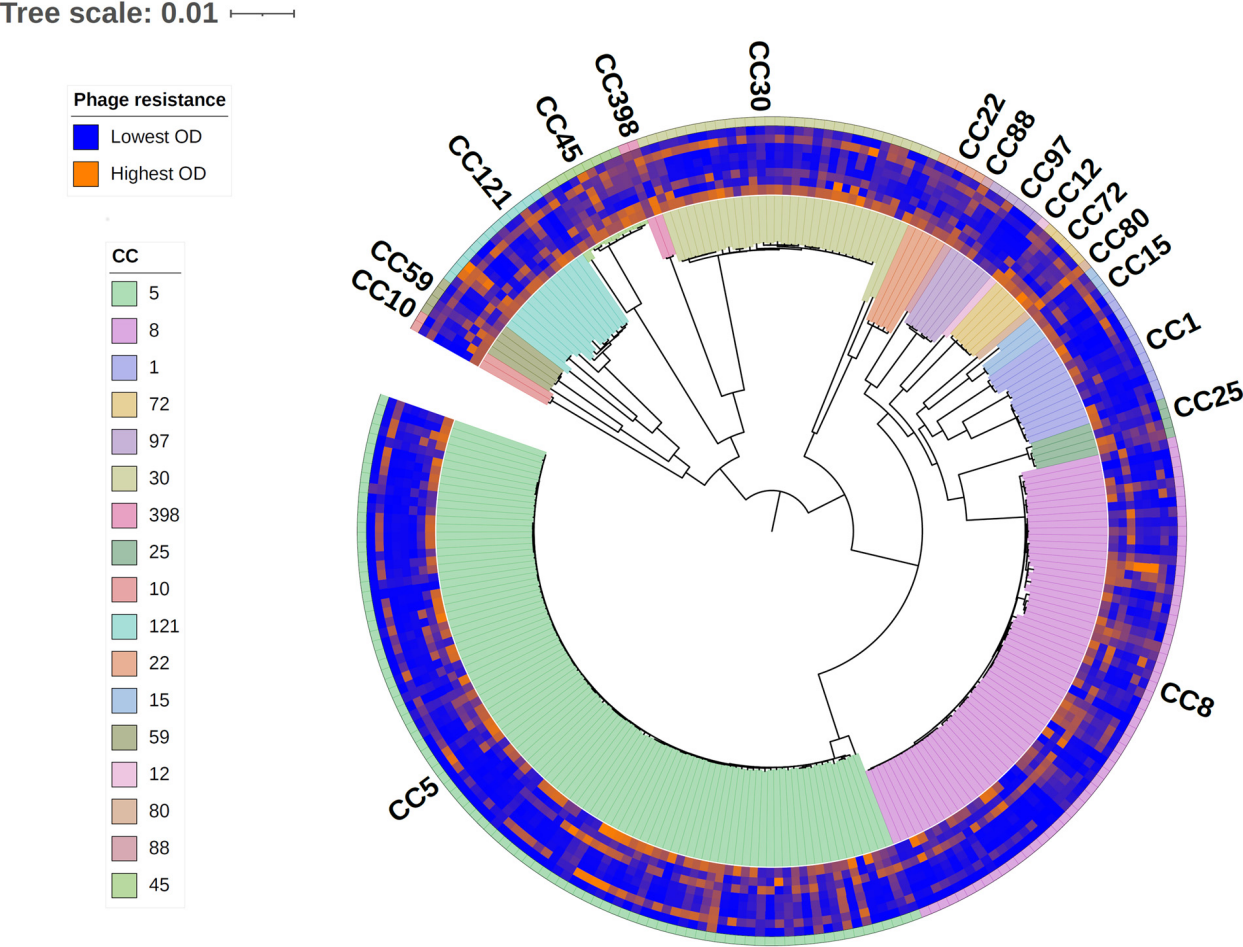
Phage resistance across the S. aureus species. Average high-throughput phage host range assay phenotypes (of at least six replicates) and corresponding strain clonal complexes were placed on a maximum-likelihood, midpoint-rooted core genome phylogeny and visualized with the Interactive Tree of Life (iTOL) (107). Phenotypes are presented on a scale from blue (lowest OD_600_, most sensitive) to orange (highest OD_600_, most resistant). Phenotypes from inside to outside correspond to phages p0045, p0006, p0017, p0017S, p002y, p003p, p0040, and pyo. CCs are shaded inside and outside the circumference of the tree.

Resistance to each phage is homoplasious, emerging independently in multiple CCs (Fig. 4). We estimated phylogenetic signal by calculating Moran’s *I*, Abouheif’s *C*_mean_, Pagel’s λ, and Blomberg’s *K* (41) for each phage host range phenotype, which resulted in statistically significant values in every case (Table 2). Both Moran’s *I* and Abouheif’s *C*_mean_ values fell between 0.17 and 0.37. Pagel’s λ values all were nearly 1, while Blomberg’s *K* values approached 0. Pagel’s λ values around 1 and Moran’s *I*/Abouheif’s *C*_mean_ values around 0 support a Brownian motion model (the phylogeny structure alone best explains the trait distribution), but Blomberg’s *K* values around 0 suggest that trait variance at the tips is greater than that predicted by the phylogeny under a Brownian motion model. All calculated phylogenetic signal values were statistically significant (*P* < 0.05 for randomization tests based on 999 simulations). Taken together, these results suggest that the structure of the phylogeny might explain the host ranges of the tested phage as expected under a Brownian motion model (random distribution of phenotypes among strains directed by the phylogeny overall). This neutral phylogenetic signal agrees with the previous finding that CC is associated with host range (Fig. 3A; see Table S4). While there is a CC association with host range, strain-specific effects may be even stronger than CC-specific effects, resulting in weak net phylogenetic signals.

**TABLE 2:** Measures of phylogenetic signal for each phage resistance phenotype^*a*^

Phage	Moran’s *I*	Abouheif’s *C*_mean_	Pagel’s λ	Blomberg’s *K*
p0045	**0.23**	**0.23**	**1.00**	**0.005**
p0006	**0.17**	**0.17**	**1.00**	**0.008**
p0017S	**0.32**	**0.32**	**1.00**	**0.007**
p002y	**0.23**	**0.23**	**1.00**	**0.008**
p003p	**0.30**	**0.30**	**1.00**	**0.012**
p0040	**0.28**	**0.28**	**1.00**	**0.014**
pyo	**0.36**	**0.37**	**1.00**	**0.006**
p0017	**0.31**	**0.31**	**1.00**	**0.014**

a Values that are significant are shown in bold. Significance was determined for 999 random permutations of the data.

### GWAS reveals novel genetic determinants of host range.

We used the GWAS tools pyseer (42) and treeWAS (43) to identify genetic loci strongly associated with the phage host range phenotype (see Fig. S3A in the supplemental material; Table 3). We chose these tools because they represent two alternatives for population structure correction: identifying principal components of a distance matrix (pyseer) and testing against phenotypes simulated based on the phylogeny (treeWAS). pyseer identified clusters of orthologous genes (COGs), SNPs, and k-mers beyond the respective multiple-corrected significance thresholds in all phages. Most phages lacked k-mer *P* value inflation, with the exceptions of p0017S, p002y, and p003p, based on associated Q-Q plots (scatter above the diagonal at *P* values of 1e−2 or more indicated *P* value inflation) (Fig. S2). The number of significant COGs detected ranged from 48 (p0017S) to 347 (pyo). Significant SNPs were detected for all phages but p0045 and p0017S and ranged from 1 (p0017) to 249 (pyo). Significant SNPs were identified in *tarJ* (pyo, 672A>G synonymous) and *tagH* (p002y, 848T>C missense and 873A>T missense; pyo, 848T>C missense, 873A>T missense, and 876C>T synonymous). TarJ is responsible for activating ribitol phosphate with CTP to form CDP-ribitol (44), while TagH is a component of the ABC transporter that exports WTA to the cell surface (9). A substantial number of the significant p0017 k-mers [1,382; −log(*P* value) = 12.259] mapped to the recently discovered host range factor *tarP*. TarP was shown to confer podovirus resistance by transferring *N*-acetylglucosamine to the C-3 position of ribitol phosphate (14). Significant k-mers also mapped to *hsdS* [32 for p002y, −log(*P* value) = 9.33; 6 for p003p, −log(*P* value) = 8.54], *oatA* [2 for p002y, −log(*P* value) = 7.75; 3 for p003p, −log(*P* value) = 8.45], and *tagH* [11 for p002y, −log(*P* value) = 9.47; 10 for p003p, −log(*P* value) = 8.81]. HsdS determines the sequence specificity of SauI restriction-modification (37), while OatA, or peptidoglycan *O*-acetyltransferase, is required for phage adsorption at least in S. aureus strain H (45). Prophage-associated genes [186 k-mers for phage tail fiber gene SRX477019_02350 for phage p0045, −log(*P* value) = 12.21; 37 k-mers for the same gene for p0040, −log(*P* value) = 8.69] were the most significantly associated with two of the tested *Siphoviridae*, i.e., phages p0045 and p0040. This result agrees with the known temperate phage resistance mechanism of superinfection immunity, in which prophages express a repressor gene that prevents transcription of lytic genes of superinfecting phages (46).

**TABLE 3:** GWAS summary statistics for each associated genetic element

Phage	No. of unique genetic elements^*a*^							
	p0045	p0006	p0017	p0017S	p002y	p003p	p0040	Pyo
COGs (pyseer)	131	49	76	48	163	175	163	347
SNPs (pyseer)	0	27	1	0	134	48	6	249
k-mers (pyseer)	820	18	7,078	101	1,734	866	180	14
SNPs (treeWAS)	0	1	0	0	0	1	1	4

a Each value represents the number of unique genetic elements of a particular type found to be significantly associated with the phage host range phenotype.

10.1128/mSphere.01263-20.1 FIG S1 Screen plot used to pick the number of dimensions for multidimensional scaling (MDS) in pyseer COG significance analysis. The number of dimensions (PCs) picked was the least possible (42), after which the eigenvalue stabilized with respect to dimension number. Download FIG S1, TIF file, 0.2 MB. Copyright © 2021 Moller et al. 2021 Moller et al. This content is distributed under the terms of the Creative Commons Attribution 4.0 International license.

10.1128/mSphere.01263-20.2 FIG S2 pyseer k-mer Q-Q plots for each phage (p0045, p0006, p0017, p0017S, p002y, p003p, p0040, and pyo). The observed *P* values were plotted relative to the expected *P* values based on the null distribution. Expected *P* values were plotted with a 95% confidence interval on the diagonal. Deviation of the observed/expected curve from the diagonal indicated *P* value inflation. Download FIG S2, TIF file, 0.9 MB. Copyright © 2021 Moller et al. 2021 Moller et al. This content is distributed under the terms of the Creative Commons Attribution 4.0 International license.

10.1128/mSphere.01263-20.3 FIG S3 GWAS approach and significant SNP annotations. (A) Overview of the genome-wide association study (GWAS) workflow. pyseer (42) associated intermediate-frequency COGs, core genome SNPs, and k-mers with each host range phenotype, while treeWAS (43) only associated core genome SNPs with each host range phenotype. SnpEff (112) classified mutation effects (synonymous, missense, or nonsense) from the corresponding Roary (102) gene sequence, while STRING (47) identified putative protein-protein interactions and PANTHER (48) identified enriched functions from lists of genes corresponding to each significant SNP or k-mer. (B) Classification of significantly associated pyseer or treeWAS SNPs based on mutational effect (synonymous, missense, or nonsense). SnpEff annotated SNP effects based on corresponding genes identified in the tested strains’ core genome with Roary. Phage 0045 was not included, as no significant SNPs were detected for its host range phenotype. *Siphoviridae* are listed in red, *Myoviridae* in blue, and *Podoviridae* in purple. Download FIG S3, TIF file, 0.8 MB. Copyright © 2021 Moller et al. 2021 Moller et al. This content is distributed under the terms of the Creative Commons Attribution 4.0 International license.

treeWAS detected 4 or fewer significant SNPs for three phages and none for phages p0045, p0017, p0017S, and p002y. Among significant SNPs, the majority were synonymous for each phage, with the exception of phage p0040 (Fig. S3B). A single nonsense mutation was detected for phage p002y. The number of significant k-mers in or near a gene detected ranged from 14 (pyo) to 7,078 (p0017).

Searches using the entire set of GWAS loci for potential enriched protein-protein interactions and pathways in the STRING (47) and Gene Ontology (48) databases (using the PANTHER tool) (see Fig. S3A in the supplemental material; see Table S5 at https://figshare.com/articles/dataset/Supplemental_Table_S5/13355945, Table S6 at https://figshare.com/articles/dataset/Supplemental_Table_S6/13355948, and Table S7 at https://figshare.com/articles/dataset/Supplemental_Table_S7/13355951) resulted in a biologically diverse group of functions. These included periplasmic substrate binding (p0017S, STRING), type I restriction-modification specificity (p0017S, STRING), metal ion binding (p002y, STRING; pyo, STRING and PANTHER), ATP binding (p002y, STRING and PANTHER; pyo, STRING), amino acid metabolism (pyo, STRING and PANTHER), pyrimidine metabolism (pyo, STRING), and RNA metabolism (p0045, PANTHER). We note that the search results are limited to genes present in NCTC 8325 and must be interpreted accordingly.

### Confirmation of causal roles for novel determinants of host range.

We next used molecular genetic experiments to confirm a causal role for genes discovered in the GWAS for which there were no previous references in the literature for a role in S. aureus phage host range. The genes (*trpA*, p002y/pyseer; *phoR*, p002y, p003p, p0040/pyseer; *isdB*, p002y, p0040/pyseer; *sodM*, p002y, p003p/pyseer; *mprF/fmtC*, p002y/pyseer; and *relA*, p003p/pyseer) were selected for validation because there were available transposon mutants in the Nebraska Transposon Mutant Library (NTML) (49) and these mutants could be backcrossed into the wild-type USA300 to eliminate second site mutations. We thus could not use transposon mutants that would confer full resistance (e.g., insertions in wall teichoic biosynthesis genes *tarJ* or *tagH*), as this resistance to phage infection would prevent lysate preparation for backcrossing. Nonetheless, we backcrossed selected mutants into their isogenic background USA300 JE2 and complemented these strains with the multicopy vector pOS1-P*lgt* (50).

**FIG 5:**
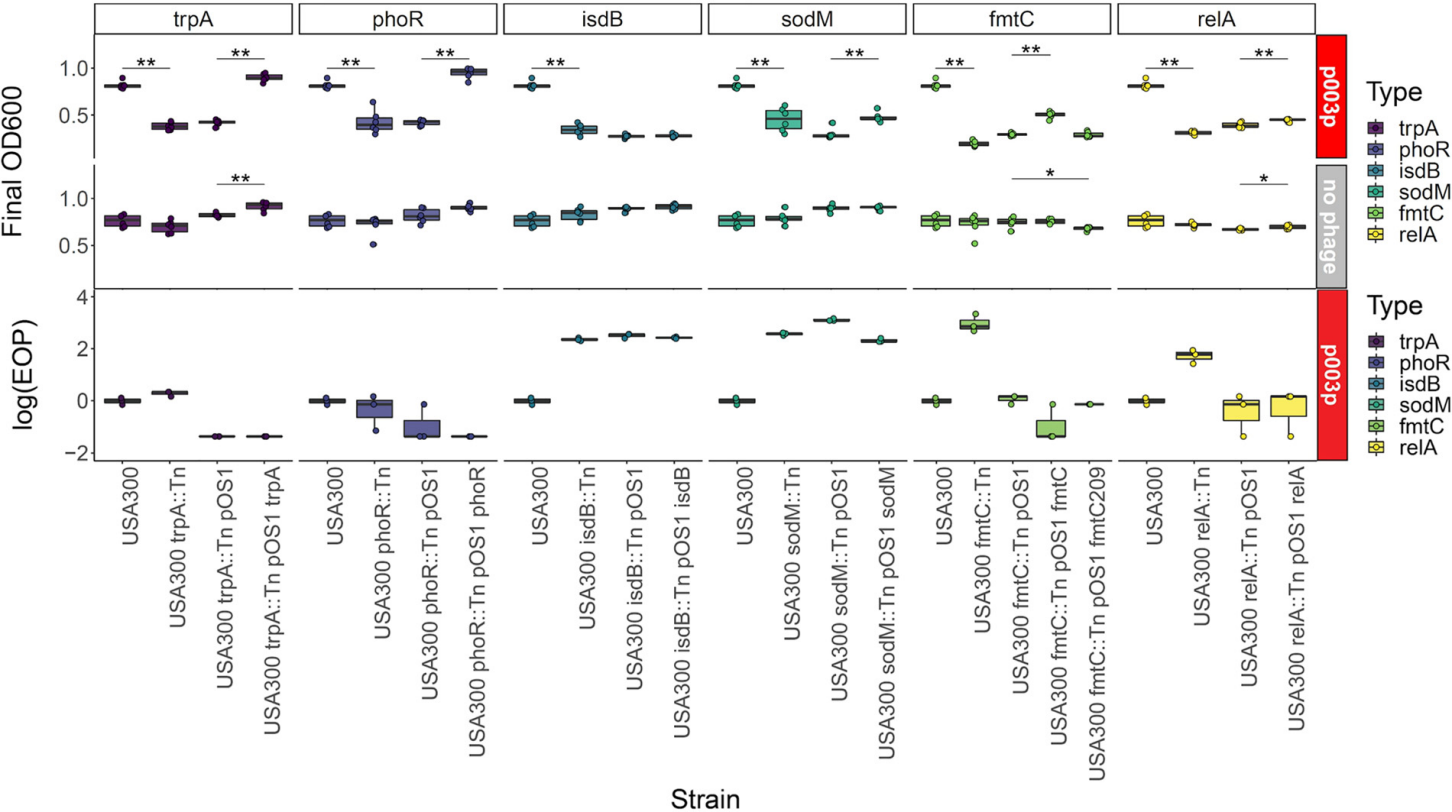
Molecular genetics validates putative phage resistance determinants. High-throughput host range assay (top) and efficiency of plating (EOP) (bottom) phenotypes demonstrating genetic validation of novel GWAS phage host range determinants are shown. Results are grouped by gene (*trpA*, *phoR*, *isdB*, *sodM*, *fmtC*, and *relA*). All assays were performed with siphovirus p003p or no phage. Each gene group includes four strains demonstrating complementation with proper controls (USA300, USA300 transposon mutant, USA300 transposon mutant with empty pOS1 vector, and USA300 transposon mutant complemented with gene in pOS1 vector). All significant (*P* < 0.05) pairwise differences (Wilcoxon signed-rank test) are indicated at the top of the corresponding box plots (ns, not significant; *, *P* = 0.01 to 0.05; **, *P* = 0.001 to 0.01; ***, *P* = 0.0001 to 0.001; ****, *P* = 0 to 0.0001).

We assessed the USA300 JE2 background, transposon mutants, transposon mutants with empty vectors, and complemented transposon mutants for growth defects and phage resistance with the previously described high-throughput (Fig. 5; see Fig. S6 in the supplemental material) and efficiency of plating (EOP) assays (Fig. 5 and Fig. S7), respectively. No strains had growth defects with respect to each other or the wild-type background (Fig. S4). We found significant decreases in phage resistance for all mutants in the presence of phages p0006, p0017S, p003p, and p0040 (*P* < 0.05, Wilcoxon signed-rank test). However, when we attempted to rescue the phenotype by complementation, we found only corresponding rescue of phage resistance back toward the wild-type phenotype in *trpA*, *phoR*, *sodM*, and *fmtC* (*P* < 0.05, Wilcoxon signed-rank test). Interestingly, the *fmtC* allele from NRS209 did not complement the *fmtC*::Tn insertion, while the *fmtC* allele from the same strain (USA300 JE2) did, suggesting allele specificity for *fmtC* in phage resistance effects. As found in growth curves (Fig. S4), in the high-throughput assay, for the most part, mutations and plasmids did not affect bacterial growth in the absence of phage (Fig. 5, no-phage panel; Fig. S6). We further evaluated bacterial survival after the high-throughput assay by measuring the number of CFU in assay soft agar after overnight culture for the *trpA* set of strains and phage p003p. As expected, the number of surviving CFU correlated with final OD, with significantly (*P* < 0.05, Wilcoxon signed-rank test) higher number of CFU and OD for USA300 JE2 than USA300 *trpA*::Tn and for USA300 *trpA*::Tn pOS1 *trpA* than USA300 *trpA*::Tn pOS1 (Fig. S5).

10.1128/mSphere.01263-20.4 FIG S4 Growth curves of USA300, USA300 transposon mutants (A), transposon mutants electroporated with the empty pOS1 vector (B), and transposon mutants complemented with vectors containing the respective genes (C) (*trpA*, *phoR*, *isdB*, *sodM*, *fmtC*, and *relA*). Strains were inoculated with a 96-pin replicator from arrayed frozen glycerol stocks into 96-well plates containing 200 μl LB-TSB 2:1 with 5 mM CaCl_2_ or the same medium supplemented with 10 μg/ml chloramphenicol in each well. We then diluted each culture 1:100 in fresh LB-TSB 2:1 with 5 mM CaCl_2_ or the same medium supplemented with 10 μg/ml chloramphenicol and collected growth curves on a BioTek Eon plate reader (37°C, 225 rpm agitation, OD_600_ measured every 10 min). Download FIG S4, TIF file, 1.4 MB. Copyright © 2021 Moller et al. 2021 Moller et al. This content is distributed under the terms of the Creative Commons Attribution 4.0 International license.

10.1128/mSphere.01263-20.5 FIG S5 Bacterial survival after completion of the high-throughput host range assay (p003p against *trpA* strains). The high-throughput assay was performed for six biological replicates of USA300, USA300 *trpA*::Tn, USA300 *trpA*::Tn pOS1, and USA300 *trpA*::Tn pOS1 *trpA* strains. (A) ODs were measured for the high-throughput phage host range assay replicates as described previously. (B) Agar plugs were removed with toothpicks and transferred to 0.8-ml volumes of sterile TMG, and bacteria were resuspended by vortexing. The resuspensions were serially diluted in TMG, and 4 μl of 1e−1 through 1e−6 dilutions were spotted four times on TSA plates. Dilution plates were grown overnight at 37°C, and colonies were counted the following day to determine the number of surviving CFU under each condition. Download FIG S5, TIF file, 0.4 MB. Copyright © 2021 Moller et al. 2021 Moller et al. This content is distributed under the terms of the Creative Commons Attribution 4.0 International license.

10.1128/mSphere.01263-20.6 FIG S6 High-throughput host range assay phenotypes demonstrating genetic validation of novel GWAS phage host range determinants. Results are grouped by gene (*trpA*, *phoR*, *isdB*, *sodM*, *fmtC*, and *relA*) and phage (p0045, p0017S, p003p, p0040, p0006, p002y, pyo, and no phage). Each group includes four strains demonstrating complementation with proper controls (USA300, USA300 transposon mutant, USA300 transposon mutant with empty pOS1 vector, and USA300 transposon mutant complemented with gene in pOS1 vector). All significant (*P* < 0.05) pairwise differences (Wilcoxon signed-rank test) are shown at the top of the corresponding box plots. *Siphoviridae* are listed in red, *Myoviridae* in blue, and the no-phage control in gray. Download FIG S6, PDF file, 0.1 MB. Copyright © 2021 Moller et al. 2021 Moller et al. This content is distributed under the terms of the Creative Commons Attribution 4.0 International license.

10.1128/mSphere.01263-20.7 FIG S7 Efficiency of plating (EOP) phenotypes demonstrating genetic validation of phage host range determinants. Undiluted through 1e−8 dilutions of phage were spotted (4 μl) three times on each top agar lawn, allowed to dry, and incubated face up overnight at 37°C, and plaques were counted at the lowest countable dilution. EOP was calculated relative to the average PFU/ml for the control strain, USA300 JE2. Results are grouped by gene (*trpA*, *phoR*, *isdB*, *sodM*, *fmtC*, and *relA*) and phage (p0045, p0017S, p003p, p0040, p0006, p002y, and pyo). *Siphoviridae* are listed in red, and *Myoviridae* in blue. Each group includes four strains demonstrating complementation with controls (USA300, USA300 transposon mutant, USA300 transposon mutant with empty pOS1 vector, and USA300 transposon mutant complemented with gene in pOS1 vector). All significant (*P* < 0.05) pairwise differences (Wilcoxon signed-rank test) are shown at the top of the corresponding boxplots. Download FIG S7, PDF file, 0.04 MB. Copyright © 2021 Moller et al. 2021 Moller et al. This content is distributed under the terms of the Creative Commons Attribution 4.0 International license.

We did not observe any significant changes in phage propagation efficiency when performing the efficiency of plating (EOP) assay on these strains, except for USA300/USA300 *trpA*::Tn pOS1 *trpA*, USA300/USA300 *phoR*::Tn pOS1, and USA300/USA300 *relA*::Tn pOS1 *relA* (*P* < 0.05, Wilcoxon signed-rank test). EOP measures differences in plaquing or actual infection and phage propagation. The growth-based assay measures survival despite infection. We interpreted the different results between the EOP and growth assays to indicate that these genes (*trpA*, *phoR*, *isdB*, *sodM*, *fmtC*, and *relA*)mostly influence survival postinfection and do not necessarily prevent infection. Taken together, these results confirmed that at least six GWAS-significant genes are implicated in phage resistance for some of the eight phages but not necessarily at the level of direct interference with phage propagation.

### Host range predictive models based on significant genetic determinants explain most phenotypic variation.

**FIG 6:**
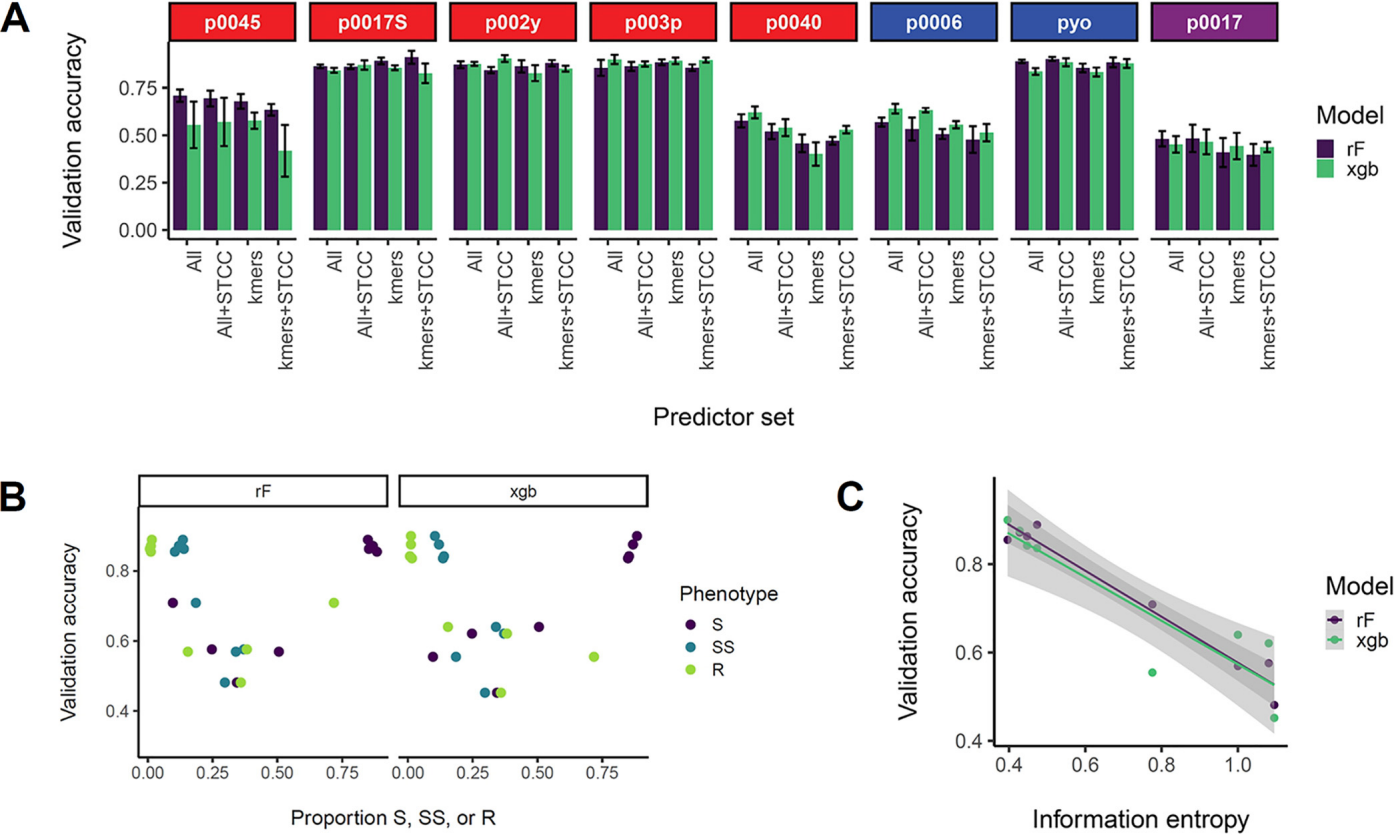
Construction of predictive models for each ternary phage resistance phenotype. Quantitative host range phenotypes were classified as sensitive (S), semisensitive (SS), or resistant (R) based on the bins (OD_600_, 0.1 to 0.4, 0.4 to 0.7, and 0.7 or more, respectively). *Siphoviridae* are listed in red, *Myoviridae* in blue, and *Podoviridae* in purple. (A) Tenfold cross-validation predictive accuracies for each phage based on two model building methods (randomForest and XGBoost) and four sets of predictors, all significant GWAS genetic determinants (COGs, SNPs, and k-mers) for a particular phage, all determinants plus corresponding strain sequence type and clonal complex (ST and CC), significant k-mers for a particular phage, and significant k-mers plus strain ST and CC. Average accuracies of four 10-fold cross-validation (CV) replicates are presented with 1 standard error above and below the mean. Validation accuracy represents the proportion of correctly identified ternary phenotypes in the validation set (one-tenth of the strain set). (B) Average accuracies from four 10-fold CV replicates for each model building method and all significant GWAS determinants as predictors relative to the proportion of each ternary phenotype (S, SS, or R) among tested strains for the corresponding phage. Three points are shown for each validation accuracy result (corresponding to each of the three possible phenotypes). (C) Average accuracies from four 10-fold CV replicates for each model building method and all significant GWAS determinants as predictors relative to the information entropy for each host range phenotype, which was calculated as described in Materials and Methods. Information entropy was calculated with a natural logarithm in natural units (nats).

In order to determine the extent to which host range is predictable by the loci identified by GWAS, we constructed predictive models for qualitative host range phenotypes using random forests, gradient-boosted decision trees, and neural networks. We determined predictive accuracy for each phage host range phenotype and four different sets of predictors (presence/absence of significant genetic determinants or k-mers from GWAS result, with or without sequence type and clonal complex for corresponding strains) with 10-fold cross-validation (Fig. 6A; Fig. S8A). In no cases were there significant differences in 10-fold cross-validation predictive accuracies between model construction methods or predictor sets used, suggesting that no combination of method and predictors improved model predictive accuracy relative to another and that there is a limit to the amount of host range variation explained by the predictive models. The phages p0017S (predictive accuracy, 0.83 to 0.87), p002y (0.81 to 0.88), p003p (0.83 to 0.92), and pyo (0.83 to 0.91) had the highest average predictive accuracies, followed by p0045 (0.67 to 0.73), p0006 (0.47 to 0.61), p0040 (0.42 to 0.61), and p0017 (0.45 to 0.54). We hypothesized that predictive accuracy correlated with host range distribution, expecting simpler distributions to be easier to predict and thus to have higher predictive accuracies. We thus examined the relationship between information entropy (average level of uncertainty or information in a variable’s possible outcomes) and predictive accuracy (Fig. 6B and C; Fig. S8B and C). We found that predictive accuracy increased at the extremes of phenotype proportion (S, SS, R) and that information entropy was negatively correlated with predictive accuracy for all models.

10.1128/mSphere.01263-20.8 FIG S8 Construction of neural network predictive models for each ternary phage resistance phenotype. Quantitative host range phenotypes were classified as sensitive (S), semisensitive (SS), or resistant (R) based on the bins (OD_600_, 0.1 to 0.4, 0.4 to 0.7, and 0.7 or more, respectively). Data preprocessing included oversampling (p0045, p0017S, p002y, p003p, or pyo), lasso regression (p0017), both (p0006), or neither (p0040). (A) Predictive accuracies for each phage based on neural networks and four sets of predictors: all significant GWAS genetic determinants (COGs, SNPs, and k-mers) for a particular phage, all determinants plus corresponding strain sequence type and clonal complex (ST and CC), significant k-mers for a particular phage, and significant k-mers plus strain ST and CC. Average accuracies of four replicates are presented with 1 standard error above and below the mean. Validation accuracy represents the proportion of correctly identified ternary phenotypes in the validation set (30% of the strain set). (B) Average accuracies from four replicates and all significant GWAS determinants as predictors relative to the proportion of each ternary phenotype (S, SS, or R) among tested strains for the corresponding phage. Three points on the same horizontal are shown for each validation accuracy result (corresponding to each of the three possible phenotypes). (C) Average accuracies from four replicates and all significant GWAS determinants as predictors relative to the information entropy for each host range phenotype, which was calculated as described in Materials and Methods. Information entropy was calculated with a natural logarithm in natural units (nats). *Siphoviridae* are listed in red, *Myoviridae* in blue, and *Podoviridae* in purple. Download FIG S8, TIF file, 1.0 MB. Copyright © 2021 Moller et al. 2021 Moller et al. This content is distributed under the terms of the Creative Commons Attribution 4.0 International license.

We also performed the same analyses on another predictive model statistic, the receiver operating characteristic (ROC) curve area under the curve (AUC), which measures the ability of the model to distinguish between classes (true positive and true negative). We found that gradient-boosted decision tree AUCs held uniform among phages, while random forest and neural network AUCs negatively correlated with information entropy (Fig. S9 and S10), suggesting that phenotype complexity (entropy) did not affect the robustness of gradient-boosted decision tree prediction. Taken together, these results show that significant GWAS determinants from this study do not completely predict phage host range and that prediction is most effective for low-complexity host range distributions, at least for random forest and neural network models.

10.1128/mSphere.01263-20.9 FIG S9 Evaluation of ternary phage resistance phenotype predictive models through receiver operating characteristic (ROC) area under the curve (AUC). Quantitative host range phenotypes were classified as sensitive (S), semisensitive (SS), or resistant (R) based on the bins 0.1 to 0.4, 0.4 to 0.7, and 0.7 or more (OD_600_), respectively. Data preprocessing included oversampling (p0045, p0017S, p002y, p003p, or pyo), lasso regression (p0017), both (p0006), or neither (p0040). (A) Tenfold cross-validation ROC AUCs for each phage based on two model building methods (randomForest and XGBoost) and four sets of predictors: all significant GWAS genetic determinants (COGs, SNPs, and k-mers) for a particular phage, all determinants plus corresponding strain sequence type and clonal complex (ST and CC), significant k-mers for a particular phage, and significant k-mers plus strain ST and CC. Average ROC AUCs of four 10-fold CV replicates are presented with q standard error above and below the mean. (B) Average ROC AUCs from four 10-fold CV replicates for each model building method and all significant GWAS determinants as predictors relative to the proportion of each ternary phenotype (S, SS, or R) among tested strains for the corresponding phage. Three points are shown for each ROC AUC (corresponding to each of the three possible phenotypes). (C) Average ROC AUCs from four 10-fold CV replicates for each model building method and all significant GWAS determinants as predictors relative to the information entropy for each host range phenotype, which was calculated as described in Materials and Methods. Information entropy was calculated with a natural logarithm in natural units (nats). *Siphoviridae* are listed in red, *Myoviridae* in blue, and *Podoviridae* in purple. Download FIG S9, TIF file, 1.5 MB. Copyright © 2021 Moller et al. 2021 Moller et al. This content is distributed under the terms of the Creative Commons Attribution 4.0 International license.

10.1128/mSphere.01263-20.10 FIG S10 Evaluation of ternary phage resistance phenotype neural network predictive models through receiver operating characteristic (ROC) area under the curve (AUC). Quantitative host range phenotypes were classified as sensitive (S), semisensitive (SS), or resistant (R) based on the bins 0.1 to 0.4, 0.4 to 0.7, and 0.7 or more (OD_600_), respectively. (A) ROC AUCs for each phage based on neural network models and four sets of predictors: all significant GWAS genetic determinants (COGs, SNPs, and k-mers) for a particular phage, all determinants plus corresponding strain sequence type and clonal complex (ST and CC), significant k-mers for a particular phage, and significant k-mers plus strain ST and CC. Average ROC AUCs of four replicates are presented with 1 standard error above and below the mean. (B) Average ROC AUCs from four replicates and all significant GWAS determinants as predictors relative to the proportion of each ternary phenotype (S, SS, or R) among tested strains for the corresponding phage. Three points are shown for each ROC AUC (corresponding to each of the three possible phenotypes). (C) Average ROC AUCs from four replicates and all significant GWAS determinants as predictors relative to the information entropy for each host range phenotype, which was calculated as described in Materials and Methods. Information entropy was calculated with a natural logarithm in natural units (nats). *Siphoviridae* are listed in red, *Myoviridae* in blue, and *Podoviridae* in purple. Download FIG S10, TIF file, 1.0 MB. Copyright © 2021 Moller et al. 2021 Moller et al. This content is distributed under the terms of the Creative Commons Attribution 4.0 International license.

## DISCUSSION

Through GWAS using a diverse natural set of S. aureus strains, we discovered numerous genetic determinants of phage host range, many of which had not been reported previously in the scientific literature. This study uses a far more diverse set of strains than the previous hypothesis-free study of S. aureus phage host range (36). However, our set of genetic loci still only partially explains the variation in the overall broad host ranges of our tested phages, as the predictive modeling results indicate.

We found that knockouts of six GWAS-significant genes. i.e., *trpA*, *phoR*, *isdB*, *sodM*, *fmtC*, and *relA*, increased phage sensitivity, suggesting that these could be targets for phage therapy adjunctive drugs. *trpA* together with *trpB* (encoding tryptophan synthase alpha and beta chains, respectively) carries out the last step in l-tryptophan biosynthesis (51). The enzymes convert indole-glycerol phosphate and serine to tryptophan and glyceraldehyde 3-phosphate (51). TrpA inactivation might then sensitize S. aureus to phage infection by increasing indole-glycerol phosphate levels at the expense of tryptophan. In the absence of *trpA*, built-up tryptophan biosynthesis intermediates including indole-glycerol phosphate may sensitize cells to phage infection, making *trpA* necessary for resistance. Alternatively, by reducing the total tryptophan pool, removing tryptophan biosynthesis may increase the proportion of tryptophan used to translate phage relative to host proteins, thus enhancing phage infection at the cost of host growth. Indeed, it is already known that throttling down protein synthesis with sublethal doses of ribosomal active antibiotics enhances plaque formation on MRSA lawns (52).

The PhoPR two-component system is responsible for regulating expression of phosphate uptake systems (ABC transporters) based on phosphate levels. In S. aureus, PhoPR is necessary for growth under phosphate-limiting conditions by regulating either phosphate transporters or other factors, depending on the environment (53). In Bacillus subtilis, the sensor kinase PhoR senses phosphate limitation through wall teichoic acid (WTA) intermediates (54) and correspondingly represses WTA biosynthesis gene expression (55). PhoPR also upregulates glycerol phosphate WTA degradation in S. aureus and B. subtilis to scavenge phosphate (56, 57). If all these mechanisms are present in S. aureus, and if there is also a pathway for degrading S. aureus ribitol phosphate (Rbo-P) WTA, PhoR activity may lead to reduced WTA under phosphate starvation, thus forming phage-resistant cells. On the other hand, as for *trpA*, *phoR* might simply be required for properly inducing the phosphate uptake necessary for survival during phage infection.

Superoxide dismutase (SodM) and phosphatidylglycerol lysyltransferase/multiple peptide resistance factor (FmtC/MprF) more likely have direct mechanistic roles in the phage infection process. SodM may be required for tolerance to cell wall stress imposed by phage infection. SodM is a Mn/Fe-dependent superoxide dismutase that converts superoxide into hydrogen peroxide and oxygen. Previous studies have shown that superoxide dismutase has affected tolerance to cell wall active antibiotics in S. aureus and Enterococcus faecalis (58, 59) and phage plaquing in Campylobacter jejuni (60). Superoxide dismutase transcripts were found to be upregulated upon phage infection in E. faecalis (61). FmtC, on the other hand, may affect the lysis step by altering cell surface charge. FmtC (MprF) alters cell surface charge first by attaching the positively charged lysine to phosphatidylglycerol through esterification with glycerol (62, 63). It then flips these modified phospholipids from the inner to the outer leaflet of the cell membrane (64). This resulting positive charge on the outer membrane confers resistance to cationic antimicrobial peptides (CAMPs) but may also alter lysis. Phage lysis depends on holin proteins, which form pores in the membrane that dissipate proton motive force and release endolysins to degrade the cell wall peptidoglycan (65–67). Because FmtC alters cell surface charge, it also could affect holin-dependent membrane depolarization, endolysin activity, or phage attachment, especially if the phage receptor-binding protein is positively charged. Interestingly enough, the *fmtC* allele from NRS209 did not complement the transposon insertion in USA300 JE2. This could indicate either a loss of function in the allele or incompatibility with some aspect of the USA300 JE2 strain.

Two of the six validated genes did not restore wild-type phenotypes upon complementation (*isdB* and *relA*). RelA, or the *relA*/*spoT* homolog in S. aureus, synthesizes (p)ppGpp in response to sensing uncharged tRNAs on the ribosome (68). Transcriptomic studies indicated that S. aureus upregulates its *relA/spoT* homolog in response to lytic phage predation (69). RelA may contribute to phage-resistant, slow-growing cell (persister) formation (70), although studies indicate that ATP depletion rather than (p)ppGpp synthesis accounts for persistence in S. aureus (71). IsdB, on the other hand, is part of the iron-regulated surface determinant (Isd) system responsible for scavenging iron from hemoglobin (72). As experiments were conducted in rich medium, the hemoglobin iron-scavenging activity of IsdB does not seem relevant, but IsdB may be an abundant surface protein, implicating it in surface occlusion. Neither *isdB* nor *relA* is in an operon, at least in USA300 JE2. It might be that the native promoters are inherently stronger than the P*lgt* promoter or are strongly upregulated during phage infection, thus affecting the efficiency of complementation. We also note for all genes that there was no apparent complementation for phages p002y and pyo (Fig. S6). In the case of the latter two, the parental USA300 JE2 strain was already sensitive to those two phages.

These validated genes along with most other GWAS-detected host range factors have not been previously reported as important in S. aureus phage infection, but the GWAS did identify some known factors. Such factors included WTA biosynthesis and modification genes *tarP*, *tarJ*, and *tagH*. While TarJ and TagH are involved in WTA biosynthesis, the WTA glycosyltransferase TarP was recently shown to directly confer *Podoviridae* resistance. Capsule biosynthesis (*cap8A* and *cap8I*) (73) and peptidoglycan modification (*oatA*) genes (45) encode surface-associated functions previously implicated in S. aureus phage resistance. Capsule or capsule overproduction is known to confer phage resistance in S. aureus (7, 12), while peptidoglycan O-acetyl groups are part of the phage receptor (45). Type I restriction-modification (*hsdS*) was implicated as well, and this is a well-known mechanism for suppression of infection across clonal complexes (37). Staphylococcal pathogenicity islands (SaPIs) were not implicated, most likely because these are highly specific to siphovirus helper phages, and even for possibly affected helper phages (80α), SaPI interference reduces but does not eliminate helper phage production (74). This means that our high-throughput assay may not capture SaPI-level effects, as it does not directly measure phage propagation through plaquing efficiency. CRISPRs were not significant in our study either, because these are rare in S. aureus strains (1, 20, 21).

Our study agreed with prior work demonstrating that S. aureus phages have broad host ranges (28–34). A major goal of our work was to create prototype predictive models for host range based on genome sequence. Genome-based predictions for several antibiotic resistance phenotypes have proven to be of similar accuracy to classic laboratory-based assays (75). We found that S. aureus host range prediction accuracy was 40 to 95%, depending on the phage. More strains and phages will need to be added to the host range matrix to make genomic host range prediction clinically useful. The difficulty in predicting resistance may come from the large number of genes found to influence the phenotype. Resistant strains may instead have individual, unique mechanisms or other traits that simply confer phage resistance, with the exception of superinfection immunity, in which host-encoded prophages prevent infection of a cognate temperate phage by repressing its lytic genes with their *cI* repressors (46). The two phages with the highest overall resistance (p0045 and p0040 [Fig. 2]) are temperate *Siphoviridae*. Most isolated S. aureus strains encode prophages (76), making superinfection immunity and the corresponding overall p0045 and p0040 resistance common in the tested strains.

There are limitations to performing phage host range measurement. The high-throughput assay did not measure lysis directly but also did not have the disadvantages of observer bias, low throughput, and qualitative output of the spot assay. In our host range assay, we measure the ability of the population overall to survive phage challenge, but this could also indicate the phage suppression of bacterial growth through some level of infection. Likewise, multiple possible sets of population dynamics confound the spot assay. Efficiency of plating (EOP), on the other hand, measures phage propagation efficiency directly by comparing phage titer on a permissive control strain to that on a test strain (77). Nonetheless, factors altering EOP still might affect any stage of the infection cycle, so EOP measurement does not suggest a possible phage resistance mechanism. The ambiguity of both assays suggests that examining the population dynamics of phages and identified mutants (e.g., *trpA*::Tn) during infection (i.e., adsorption rate, latent period, and burst size from a one-step growth curve) would be worthy for future studies to pinpoint the specific mechanism by which that gene affects phage resistance. We also recognize that a multitude of environmental variables (temperature, multiplicity of infection, growth medium) might influence the assay.

There are also some limitations inherent in GWAS approaches. Bacterial GWAS associates homoplasic variants that arise from parallel evolution or recombination with a phenotype of interest (78, 79). While bacterial GWAS can find more types of genetic events (either loss of function or gain of function, mutation, insertion, deletion, recombination, and so on, but not genes with no changes) and more broadly relevant genes and polymorphisms related to a phenotype than screening transposon mutants in a single genetic background, clonal population structure, abundant small effect variants, and genetic interactions hamper it (78). When recombination is relatively rare in a species, like S. aureus, large numbers of variants remain in linkage disequilibrium, making it difficult to distinguish lineage from strain-level effects. Loci linked to a causative variant may then be detected as false positives. While we have validated at least a few genes as true positives, and we expect phylogenetically hierarchical effects on host range based on reviewing past work (1), our GWAS methods also include various corrections for clonal population structure when variants are associated.

Two recent studies used single gene knockout, overexpression, and transcriptional suppression methods as well as global transcriptional profiling to identify phage resistance determinants in Escherichia coli (80) and Enterococcus faecalis (61). Unlike these previous studies, our findings are not limited to one or a few genetic backgrounds, making them more widely applicable to the species and its underlying evolution. Nonetheless, extensive functional molecular genetics studies will be needed to distinguish genes that truly contribute to host range from false positives. These studies, like those in E. coli and E. faecalis, would complement the GWAS with global searches for phage resistance genes in a single genetic background, such as transposon insertion sequencing (Tn-Seq), dual-barcoded shotgun expression library sequencing (DUB-Seq), and CRISPR interference to identify genes required for surviving phage infection and transcriptome sequencing (RNA-Seq) to identify genes differentially regulated in response to phage infection. Such work would both corroborate GWAS results and fill in the gaps, possible determinants not present or conserved in enough of the resistant or sensitive population.

Our results have important consequences for phage therapy, phage-small molecule combination therapy, and horizontal gene transfer in the species. The genes identified expand the set of potential combination therapies by providing additional targets to which to design small molecules to interfere with phage resistance. Already, combination phage-antibiotic therapies have shown promise for clearing biofilms and reducing emergence of antibiotic resistance in S. aureus (81), and ribosomal active antibiotics are known to enhance MRSA phage sensitivity at sublethal doses (52). Additionally, because the phage receptor WTA is necessary for methicillin resistance (39) and WTA inhibition resensitizes MRSA to methicillin (40), phages have the exciting possibility of inducing collateral beta-lactam sensitivity. We also cannot discount the possibility that phage resistance polymorphisms are the result of selection by other stresses besides phage infection, such as immune escape, interbacterial interactions, or antibiotic selection. Wall teichoic acid, the S. aureus phage receptor, for example, is also important for colonization, antibiotic resistance, and immune evasion (39, 82–87). Because we identified phage host range determinants, we also gained insights into the evolution of the S. aureus through horizontal gene transfer (HGT). Transduction, the transfer of host genetic material between strains by abortive phage infection, is a major mechanism of HGT (88) and recombination (89) in the species. There is a trade-off between the need to resist phage killing and the need to adapt by gaining new virulence genes (such as Panton-Valentine leukocidin) (90) through HGT. It is possible that the most transducible strains are both more sensitive to killing by phage infection and more able to outcompete other strains for advantageous genetic material. The finding that even the most resistant strains (NRS148, NRS209, and NRS255) were still sensitive to two out of the eight phages may be the result of a selection for sensitivity that could be the Achilles’ heel of S. aureus when confronted by phage therapy.

## MATERIALS AND METHODS

### Strains, media, and phage propagation.

Phages used in this study were phage p0045 (80α-like), p0017S, p002y (DI), p003p (Mourad 87), and p0040 (Mourad 2) (*Siphoviridae*); p0006 (K) and pyo (*Myoviridae*); and p0017 (HER49/p66) (*Podoviridae*). All phage genomic DNA was isolated with the bioWORLD phage DNA isolation kit by following the manufacturer’s directions after phage precipitation by a previously described protocol. The corresponding genomes were prepared for sequencing with a one-dimensional (1D) ligation sequencing kit (SQK-LSK109) or 1D rapid sequencing kit (SQK-RAD004) and sequenced with an Oxford Nanopore MinION using a Flongle flow cell (FLO-FLG001). Phages p0045, p0017S, p002y (DI), p003p (Mourad 87), p0040 (Mourad 2), and p0006 (K) genomes were also sequenced with Illumina technology by the Microbial Genome Sequencing Center (MiGS) at the University of Pittsburgh.

All *Siphoviridae* and *Myoviridae* were propagated in S. aureus RN4220, while the sole podovirus was propagated in S. aureus RN4220 *tarM*::Tn, which was constructed by transducing strain RN4220 with Nebraska Transposon Mutant Library (NTML) (49) strain USA300 JE2 *tarM*::Tn (NE611) phage 0045 lysate. Strains, phages, and plasmids used for phage propagation and molecular genetic validation of GWAS results are listed in Table 4. Transduction was performed according to a previously published protocol (91). All overnight cultures were grown in LB-Trypticase soy broth at 2:1 (LB-TSB 2:1) supplemented with 5 mM CaCl_2_ to promote phage adsorption.

**TABLE 4:** Strains, phages, and plasmids used for phage propagation and molecular genetic validation of GWAS results

Strain, phage, or plasmid	Characteristics/description	Reference(s)
E. coli strains		
DH5ɑ	E. coli cloning strain; F^−^ *endA1 glnV44 thi-1 recA1 relA1 gyrA96 deoR nupG purB20* φ80d*lacZ*ΔM15 Δ(*lacZYA*-*argF*)*U169 hsdR17* (r_K_^–^ m_K_^+^) λ^−^	119
IM08B	E. coli cloning strain with S. aureus CC8 DNA modification; DNA cytosine methyltransferase (*dcm*)-negative mutant of E. coli K-12 DH10B; *mcrA* Δ(*mrr-hsdRMS-mcrBC*) φ80*lacZ*ΔM15 Δ*lacX74 recA1 araD139* Δ(*ara-leu*)*7697 galU galK rpsL endA1 nupG* Δ*dcm* Ω*Phelp-hsdMS* (CC8-2) Ω*PN25-hsdS* (CC8-1)	114
		
S. aureus strains		
RN4220	Phage propagation strain; background for transducing *tarM*::Tn; cloning intermediate for pOS1-P*lgt*-*fmtC*	120
RN4220 *tarM*::Tn	Podovirus (p0017) propagation strain; generated by transducing RN4220 with USA300 JE2 *tarM*::Tn (NE611) phage 0045 lysate	This study
USA300 JE2	Wild-type for genetic validation experiments and background for transposon mutant backcrossing	49
USA300 JE2 *tarM*::Tn (NE611)	Transposon mutant transduced into RN4220 to make RN4220 *tarM*::Tn by a NE611 phage 0045 lysate	49
USA300 JE2 *trpA*::Tn	Mutant NE304 backcrossed into USA300 JE2	This study
USA300 JE2 *trpA*::Tn pOS1	Complemented backcrossed mutant with empty vector	This study
USA300 JE2 *trpA*::Tn pOS1 *trpA*	Complemented backcrossed mutant with *trpA* from USA300 JE2	This study
USA300 JE2 *phoR*::Tn	Mutant NE618 backcrossed into USA300 JE2	This study
USA300 JE2 *phoR*::Tn pOS1	Complemented backcrossed mutant with empty vector	This study
USA300 JE2 *phoR*::Tn pOS1 *phoR*	Complemented backcrossed mutant with *phoR* from USA300 JE2	This study
USA300 JE2 *isdB*::Tn	Mutant NE1102 backcrossed into USA300 JE2	This study
USA300 JE2 *isdB*::Tn pOS1	Complemented backcrossed mutant with empty vector	This study
USA300 JE2 *isdB*::Tn pOS1 *isdB*	Complemented backcrossed mutant with *isdB* from USA300 JE2	This study
USA300 JE2 *sodM*::Tn	Mutant NE1224 backcrossed into USA300 JE2	This study
USA300 JE2 *sodM*::Tn pOS1	Complemented backcrossed mutant with empty vector	This study
USA300 JE2 *sodM*::Tn pOS1 *sodM*	Complemented backcrossed mutant with *sodM* from USA300 JE2	This study
USA300 JE2 *fmtC*::Tn	Mutant NE1360 backcrossed into USA300 JE2	This study
USA300 JE2 *fmtC*::Tn pOS1	Complemented backcrossed mutant with empty vector	This study
USA300 JE2 *fmtC*::Tn pOS1 *fmtC*	Complemented backcrossed mutant with *fmtC* from USA300 JE2	This study
USA300 JE2 *fmtC*::Tn pOS1 *fmtC209*	Complemented backcrossed mutant with *fmtC* from NRS209	This study
USA300 JE2 *relA*::Tn	Mutant NE1714 backcrossed into USA300 JE2	This study
USA300 JE2 *relA*::Tn pOS1	Complemented backcrossed mutant with empty vector	This study
USA300 JE2 *relA*::Tn pOS1 *relA*	Complemented backcrossed mutant with *relA* from USA300 JE2	This study
		
Phages		
p0045 (80α-like)	*Siphoviridae* phage; also used for backcrossing and pOS1-P*lgt*-*fmtC* transduction from RN4220 into USA300 *fmtC*::Tn	5, 6, 121
p0006 (K)	*Myoviridae* phage; GenBank accession no. NC_005880.2	30, 31, 122
p0017 (HER49/p66)	*Podoviridae* phage; GenBank accession no. NC_007046.1	This study
p0017S	*Siphoviridae* phage	This study
p002y (DI)	*Siphoviridae* phage	This study
p003p (Mourad 87)	*Siphoviridae* phage	This study
p0040 (Mourad 2)	*Siphoviridae* phage	This study
pyo	*Myoviridae* phage; BioProject accession no. PRJNA477834	81, 123
		
Plasmids		
pOS1-P*lgt*	Empty complementation vector	50
pOS1-P*lgt*-*trpA*	Complementation vector with *trpA* cloned downstream of P*lgt*	This study
pOS1-P*lgt*-*phoR*	Complementation vector with *phoR* cloned downstream of P*lgt*	This study
pOS1-P*lgt*-*isdB*	Complementation vector with *isdB* cloned downstream of P*lgt*	This study
pOS1-P*lgt*-*sodM*	Complementation vector with *sodM* cloned downstream of P*lgt*	This study
pOS1-P*lgt*-*fmtC*	Complementation vector with *fmtC* cloned downstream of P*lgt*	This study
pOS1-P*lgt*-*fmtC209*	Complementation vector with *fmtC* from NRS209 cloned downstream of P*lgt*	This study
pOS1-P*lgt*-*relA*	Complementation vector with *relA* cloned downstream of P*lgt*	This study

Phages were propagated by inoculating a chunk of soft agar containing a plaque and surrounding bacteria into liquid medium. Phage lysates in TMG (Tris-magnesium-gelatin) buffer were spotted (4 μl) on a top agar (0.8% agar, 0.8% NaCl) lawn (5 ml) containing 0.2 ml of a 1:10 dilution of an RN4220 or RN4220 *tarM*::Tn overnight culture (18 h of growth, 37°C, 250 rpm). After overnight growth at 37°C, a chunk of soft agar containing a plaque and surrounding bacteria was inoculated into 35 ml of LB-TSB 2:1 with 5 mM CaCl_2_. This phage-bacterium coculture was grown overnight at 37°C and 250 rpm, centrifuged for 20 min at 4,000 rpm, and filtered with a 0.45-μm syringe filter before being stored at 4°C. The resulting lysate was titered on RN4220 (*Siphoviridae* or *Myoviridae*) or RN4220 *tarM*::Tn (*Podoviridae*).

### Phage resistance/host range assays.

Two hundred fifty-nine previously genome-sequenced S. aureus strains consisting of 126 strains from the Network on Antimicrobial Resistance in Staphylococcus aureus (NARSA) repository (NCBI BioProject accession no. PRJNA289526) (92), 69 strains previously sequenced in a vancomycin-intermediate S. aureus (VISA) study (93) (PRJNA239001), and 64 strains previously sequenced in a cystic fibrosis (CF) lung colonization study (94) (PRJNA480016) were rapidly profiled for resistance to the eight phages using a high-throughput assay. Arrayed glycerol (50%) stocks of the strains were used to inoculate 96-well plates containing 200 μl of LB-TSB 2:1 with 5 mM CaCl_2_ in each well using a 96-pin replicator. Cultures were grown overnight at 37°C and 225 rpm. The following day, overnight cultures were diluted 1:10 in double-distilled water (ddH_2_O). In order to permit phage adsorption, 10 μl of each phage lysate (∼1e9 PFU/ml) was coincubated with 10 μl of each overnight culture dilution for 30 min at room temperature in 96-well plates. A 200-μl volume of molten LB-TSB-CaCl_2_ agar (LB-TSB 2:1 with 5 mM CaCl_2_ and 0.4% agar) was then added to each well containing the culture-phage mixtures and allowed to solidify. After incubation overnight (37°C), the plates were photographed and final optical densities at 600 nm (OD_600_) per well were measured using a plate reader (BioTek Eon). Strains were categorized as sensitive (OD_600_, 0.1 to 0.4), semisensitive (0.4 to 0.7), or resistant (0.7 or greater) on the basis of classifying the average final OD_600_ from at least six replicates into three equal bins (with the third bin counting outlier resistant strains with OD_600_s above 1). Strains and host range phenotypes (quantitative and quantitative converted to ternary) are listed in Tables S1 and S2.

High-throughput assays were also calibrated against a standard spot assay. One hundred eight NARSA strains were tested for resistance to five of the eight phages listed previously (phages p0045, p0006, p0017S, p002y, and p003p). Briefly, an overnight culture of each strain was diluted 1:10 in ddH_2_O, and a top agar lawn (0.2 ml of dilution per 5 ml molten top agar) was poured on a Trypticase soy agar (TSA) plate. After solidification, each of the five lysates was spotted (4 μl) twice on the top agar lawn and allowed to dry. The plates were then incubated face up overnight at 37°C, and the spots were evaluated for clearing (sensitive), turbid clearing (semisensitive), or no clearing (resistant) the following day. High-throughput assay and spot assay phenotypes were compared in box plots made with ggplot2 (95). The statistical significance of high-throughput assay phage resistance differences between all possible pairs of sensitive (S), semisensitive (SS), and resistant (R) strains was assessed with Wilcoxon signed-rank tests.

### Bioinformatic processing.

Phage p0017 and pyo genomes were assembled from Oxford Nanopore reads with canu 2.0 (96). Hybrid Illumina/Nanopore phage genome assemblies were constructed using Unicycler 0.4.8, filtering for contigs with coverage higher than 5× (97). The average nucleotide identity (ANI) was then determined among all phage contigs using fastANI 1.31 (98), which is shown as a lower-triangle identity matrix in Table S1. All S. aureus genomes were processed using the Staphopia analysis pipeline (99), which included *de novo* assembly using SPAdes (100) and annotation using Prokka (101). The core genome phylogenetic tree was constructed by first determining the core genome alignment for all tested strains with Roary (102), correcting for recombination with Gubbins (103), and then generating a maximum-likelihood phylogenetic tree with IQ-TREE (104). Strains (253 in total) for which there are corresponding phage resistance phenotypes (quantitative and qualitative), BioProject, BioSample, and SRA accessions, sequence types, clonal complexes, isolation years, and isolation locations are listed in Table S2. MLST (multilocus sequence typing) sequence types were identified for each genome with the mlst command line tool (105), which uses the PubMLST website (https://pubmlst.org/) (106). Quantitative phage resistance phenotypes were annotated on the tree using the Interactive Tree of Life (iTOL) (107).

### Preliminary phenotype analysis.

Phage resistance phenotypes were initially placed on a core genome phylogenetic tree and were associated with two factors, clonal complex (CC) and MRSA/MSSA genetic background. Phage resistance associations with CC and MRSA/MSSA were visualized in box plots made with ggplot2 (95). Statistical significance of phage resistance differences between MRSA/MSSA was determined with Wilcoxon signed-rank tests. Statistical significance of overall phage resistance differences between represented CCs was determined using one-way analysis of variance (ANOVA) tests with or without phylogenetic correction.

### Measuring phylogenetic signal.

Four different measures of phylogenetic signal were calculated for each phenotype: Abouheif’s *C*_mean_, Moran’s *I*, Pagel’s λ, and Blomberg’s *K* (41). Abouheif’s *C*_mean_ and Moran’s *I* were calculated with the abouheif.moran function from the adephylo R package (108), while Pagel’s λ and Blomberg’s *K* were calculated using the phylosig function from the phytools R package (109). Phylogenetic signal was determined using the core genome phylogenetic tree annotated with quantitative phage resistance data previously described. Randomization tests for phylogenetic signal calculation were performed with 999 permutations of the data.

### GWAS.

Genotypes were associated with phage host range phenotype data using two different genome-wide association study (GWAS) pipelines, i.e., pyseer 1.2.0 (42) and treeWAS 1.0 (43). pyseer associated clusters of orthologous genes (COGs), core genome single nucleotide polymorphisms (SNPs), and k-mers with lengths between 6 and 610 bp with each phenotype, while treeWAS associated only biallelic core genome SNPs with the phenotype. treeWAS used the recombination-corrected core genome phylogeny for population structure correction, while pyseer used a conversion of the phylogeny into a kinship matrix. The core genome alignment was rearranged to set N315 as the reference (first sequence). We chose N315 as the reference because it was used as a global S. aureus reference for the Staphopia project (99). SNPs were called from the core genome alignment with Snp-sites (110). For identifying significantly associated genetic determinants, a Bonferroni correction of 0.05/6,058 or 8.25e−6 was set for COG GWAS, 0.05/15,557 or 3.21398e−6 for SNP GWAS, and 0.05/2,304,257 or 2.17e−8 for k-mer GWAS, counting the numbers of intermediate-frequency COGs, biallelic core genome SNPs, and unique k-mers, respectively, as hypotheses to be tested.

pyseer SNP and COG association analyses performed multidimensional scaling (MDS) on a Mash distance matrix between tested strains to correct for population structure. pyseer SNP association was performed with a fixed effect (for variant and covariate lineage) model, the default 10 multidimensional scaling (MDS) dimensions retained, and lineage effect testing on each quantitative phage resistance/host range phenotype for all biallelic core genome SNPs. pyseer COG association was performed with a fixed-effect model on each phenotype and nine MDS dimensions retained for intermediate frequency COGs (see Fig. S1 in the supplemental material). pyseer k-mer association was performed with a FaST-LMM linear mixed (combined fixed variant/covariate lineage and random kinship effects) model on each quantitative phenotype for unique k-mers between 6 and 610 bp in length extracted from genomes of all tested strains. pyseer k-mer association analyses used a kinship matrix between tested strains constructed from the core genome phylogeny to correct for population structure and set a minor allele frequency cutoff for analysis of 1%, like SNP and COG analyses. SNP and k-mer association *P* values were demonstrated relative to genetic coordinates using Manhattan plots (with phandango) (111). Associations for all k-mers were assessed for *P* value inflation (exceeding the observed/expected *P* value diagonal below 1e−2) using Q-Q plots (Fig. S2). Significant SNPs and k-mers were annotated using SnpEff (112) (relative to the Roary N315 core genome sequence) and downstream analysis scripts included with pyseer, respectively, identifying the genes containing the genetic elements (or near the genetic elements, in the case of k-mers) and mutation effects, in the case of SNPs.

treeWAS was performed for each phage resistance phenotype using the R package with core genome alignment, IQ-TREE core genome phylogeny, and quantitative phage resistance phenotype as inputs and with default parameters. Significant treeWAS SNPs were annotated using SnpEff (112) relative to the core genome sequence of strain N315 (113).

### Functional annotation and network analysis of significantly associated genes.

Genes with significant association from the GWAS (containing SNPs and either near to or overlapping with k-mers) were then used to identify enriched protein functions or possible protein-protein interactions. Gene name lists for each phage were converted to NCTC 8325 RefSeq protein accession lists for use with STRING (47) and PANTHER (48), which depend on NCTC 8325 S. aureus accessions. To convert genes containing significant SNPs to NCTC 8325 accessions, Roary N315 core genes were aligned against NCTC 8325 RefSeq proteins with NCBI blastx (one maximum target sequence, one maximum high scoring pair, default e-value). Gene names matching NCTC 8325 RefSeq accessions were converted for each significant SNP using these alignment results. To convert genes containing significant k-mers to NCTC 8325 accessions, all significant genes were aligned against NCTC 8325 RefSeq proteins with blastx (one maximum target sequence, one maximum high scoring pair, default e-value). Gene names matching NCTC 8325 RefSeq accessions were converted for each significant k-mer using these alignment results. Any gene names not mapped to any NCTC 8325 RefSeq protein accessions after this procedure were left unchanged. Lists of significant genes for each phage, for all phage morphological classes (*Siphoviridae*, *Myoviridae*, and *Podoviridae*), and for each life cycle type (virulent or temperate) were used as inputs for STRING and PANTHER. STRING network properties (nodes, edges, average node degree, average local clustering coefficient, expected number of edges, and protein-protein interactions (PPI) enrichment *P* value) were saved for each input, while PANTHER functional classification and statistical overrepresentation test analyses were performed for each input with respect to molecular function, biological process, cellular component, protein class, and pathway.

### Genetic validation of novel phage resistance mechanisms.

Six genes (*trpA*, *phoR*, *isdB*, *sodM*, *fmtC*, and *relA*) found to contain significantly associated SNPs or k-mers for any phage resistance phenotype were validated to cause phage resistance changes when knocked out in a single S. aureus genetic background (USA300 JE2). Transposon insertion mutants in each gene were selected from the Nebraska Transposon Mutant Library (NTML) (49) and backcrossed into USA300 JE2 through the transduction method previously described (91) to eliminate any possible secondary acquired mutations. Backcrossed mutants were then complemented with each gene cloned into the vector pOS1-P*lgt* (50). Relevant strains (selected mutants and complemented strains) are listed in Table 4. Growth curves were performed on all listed strains (Fig. S4). USA300 JE2, respective transposon mutants, empty vector controls, or complemented mutants were inoculated with a 96-pin replicator from arrayed frozen glycerol stocks into 96-well plates containing 200 μl LB-TSB 2:1 with 5 mM CaCl_2_ or the same medium supplemented with 10 μg/ml chloramphenicol in each well. We then diluted each culture 1:100 in fresh LB-TSB 2:1 with 5 mM CaCl_2_ or the same medium supplemented with 10 μg/ml chloramphenicol and collected growth curves on a BioTek Eon plate reader (37°C, 225 rpm agitation, OD_600_ measured every 10 min).

Genes were cloned into pOS1-P*lgt* either through splicing overlap extension (SOE) PCR (*trpA*, *phoR*, and *sodM*) or through NEB HiFi assembly (*isdB*, *fmtC*, and *relA*). Each gene and pOS1-P*lgt* were amplified with the primers listed in Table S3 at https://figshare.com/articles/dataset/Supplemental_Table_S3/13355939 to create overlap into the corresponding fragment by using NEB Q5 high-fidelity DNA polymerase according to the manufacturer’s directions. All genes were amplified from USA300 JE2 genomic DNA except for *fmtC*, which was amplified both from USA300 JE2 and NRS209. Genes were cloned into the same site downstream of the P*lgt* promoter. For SOE PCR, AMpure XP bead-purified gene and vector fragments were mixed together at a ratio of 1:59 and amplified for 20 cycles with NEB Q5 high-fidelity polymerase at an annealing temperature of 60°C. For HiFi assembly, purified gene and vector fragments were mixed together at a ratio of 1:2 (less than 0.2 pmol DNA total) and incubated with NEBuilder HiFi DNA assembly master mix for 3 h at 50°C. SOE PCR and HiFi assembly products were transformed into NEB DH5ɑ competent cells (high efficiency), plated on LB agar with ampicillin (100 μg/ml), and grown overnight at 37°C. Transformants were verified by colony PCR with the respective LF and RR primers listed in Table S3. Plasmids were extracted from verified transformant overnight cultures with the Promega PureYield plasmid miniprep system. These plasmids were then transformed into E. coli IM08B (114) to improve electroporation efficiency into the USA300 JE2 transposon mutants.

Electrocompetent S. aureus cells (USA300 JE2 transposon mutants) were prepared as previously described (115). S. aureus electrocompetent cells were electroporated with 2 μg of ethanol-precipitated plasmid DNA (empty vector and vector with insert corresponding to transposon insertion). Electrocompetent cells were first thawed, centrifuged, and resuspended in 50 μl 10% glycerol–0.5 M sucrose. After plasmid DNA was added, cells were transferred to 0.1-cm electroporation cuvettes and pulsed at 2.1 kV, 100 Ω, and 25 μF. Immediately after electroporation, 1 ml of TSB–0.5 M sucrose was added to the cuvette, and the culture was transferred to an Eppendorf tube to recover for 90 min at 37°C and 250 rpm. Dilutions of the outgrowth were plated on TSA with chloramphenicol (10 μg/ml) and grown overnight at 37°C. Electroporants were verified by colony PCR with the respective LF and RR primers listed in Table S3.

pOS1 *fmtC* and *relA* were introduced into USA300 JE2 transposon mutants, however, by transduction from RN4220. S. aureus RN4220 was electroporated with pOS1 *fmtC* (USA300), pOS1 *fmtC* (NRS209), and pOS1 *relA* plasmids according to the procedure described previously. Plasmids were then transduced from RN4220 to USA300 JE2 transposon mutants according to a previously published procedure (91). Briefly, a recipient strain was infected with donor phage at a multiplicity of infection (MOI) of 0.1 after supplementation with CaCl_2_. The infected culture was then outgrown in TSB supplemented with sodium citrate to prevent phage lysogeny. The outgrowth culture was plated on TSA supplemented with both chloramphenicol (10 μg/ml) and sodium citrate (40 mM) to select for plasmids and inhibit lysogeny, respectively.

Mutants and their complemented derivatives were assessed for phage resistance and host range both through the high-throughput assay described previously and the efficiency of plating (EOP) assay (77) to assess bacterial growth in the presence of phage and phage plaquing efficiency, respectively. The high-throughput host range assay was performed as described earlier, but overnight cultures of strains were grown in LB-TSB 2:1 with 5 mM CaCl_2_ supplemented with chloramphenicol (10 μg/ml) to maintain plasmid selection in the case of complemented strains for this and the EOP assay. The EOP assay was performed by spotting 4 μl of neat through 1e−8 dilutions of phages p0045, p0006, p0017, p0017S, p002y, p003p, p0040, and pyo on lawns (0.2 ml of a 1:10 overnight culture dilution mixed with 5 ml of top agar) of a test and a reference (USA300) strain. Lawns were poured on TSA plates. EOP was calculated by dividing the phage titer on the test strain by that on the reference strain.

Additional experiments with the *trpA* mutant set and phage p003p examined bacterial survival after performance of the phage-culture soft agar coincubation of the high-throughput assay. The high-throughput assay was performed as described earlier for six replicates of USA300, USA300 *trpA*::Tn, USA300 *trpA*::Tn pOS1, and USA300 *trpA*::Tn pOS1 *trpA* strains. The corresponding ODs were recorded as described for the high-throughput phage host range assay (Fig. S5A). Agar plugs were then removed with toothpicks, placed in 0.8-ml volumes of sterile TMG, and broken apart by vortexing. The resuspensions were then serially diluted in TMG, and 4-μl volumes of 1e−1 through 1e−6 dilutions were spotted four times on TSA plates. Dilution plates were grown overnight at 37°C, and colonies were counted the following day to determine the number of surviving CFU under each condition (Fig. S5B).

### Construction of phage resistance phenotype predictive models.

Phage resistance predictive models were constructed using three methods, i.e. random (decision) forests, gradient-boosted decision trees, and neural networks. Random forests were generated using the randomForest R package, and gradient-boosted decision trees were generated with the XGBoost R package (116). Ternary (S, SS, or R) phenotypes converted from the original high-throughput assay quantitative phenotypes (described in “Phage resistance/host range assays”) were set as the response variable, while either the presence or absence of each significant genetic element, each k-mer, or one of the previous two sets (all elements or just k-mers) and both strain sequence type (ST) and clonal complex (CC) were set as predictor variables. Random forest and XGBoost predictive accuracy and receiver operating characteristic (ROC) area under the curve (AUC) were determined on the validation set through multiple replicates of 10-fold cross-validation, in which alternating tenths of data are used for validation while the model is trained on the remaining data. The optimal number of rounds (iterations) for XGBoost was determined for each phage and set of input predictor variables with 5-fold cross-validation. XGBoost model training also used the softmax objective for multiclass (three classes—S, SS, and R) classification.

Neural network model construction was more complicated, as it involved a preprocessing step to balance data sets where necessary. Oversampling or a combination of over- and undersampling methods was performed to balance specific data sets. For the oversampling method, new samples of the minority classes were randomly generated with replacement so that the number of samples for each class would be equal to that of the majority class in the original data set. For the combination method, the synthetic minority oversampling technique (SMOTE) for oversampling and Tomek links for undersampling were performed together. However, for phages with limited cases for one class type, such as p002y, we could not conduct undersampling. Therefore, for such data sets, only the oversampling method was performed. The new balanced data sets were then split into training and validation sets with 30% validation. Random splits were performed four times to generate four replicates for evaluation, each with different train and test data sets. Each replicate was evaluated as described before, with validation set prediction accuracy and ROC AUC.

Neural network models were constructed three ways: (i) with or without oversampling or with an oversampling-undersampling combination alone, (ii) as in (i) but with a regularizer and dropout layer, or (iii) as in (i) but with lasso regression for feature selection. All methods used ADAM (117) for optimizing and sparse categorical cross-entropy for loss. For imbalanced data sets, the oversampling and combination over- and undersampling methods were used as well, if possible. The fully connected neural network was constructed based on the selected, balanced data set. We then found both training and prediction accuracy to evaluate performance for each network model. We note that network models were optimized for each replicate training set, which means there may be different network models for the four replicates. In the first method, fully connected neural network models were constructed on data sets either originally balanced or balanced after oversampling/combination methods, with no further correction. Since some network models have high prediction accuracies, it is possible that these models are overfitting, so the second method adds a regularizer and a dropout layer to fully connected neural networks as new models. Finally, for some network models, the prediction accuracies were not as high as others. Thus, in the third method, lasso regression was performed to select important features and improve performance. A neural network model was constructed on the new data set based on these selected features.

Information entropy was compared to average randomForest and XGBoost 10-fold cross-validation and neural network predictive accuracies and ROC AUCs after calculation by using the following equation (118), where *H* is the total information entropy, *P_x_*(*x_i_*) is the probability of event *x_i_*, *n* is the number of possible events, and the three possible events are S, SS, and R phenotypes:

$$H=-\overset{n}{\underset{i=1}{\sum }}P_{X}(x_{i})\text{ ln}\left[P_{X}\left(x_{i}\right)\right]$$
