# Supplementary figures and images for: Genes Influencing Phage Host Range in Staphylococcus aureus on a Species-Wide Scale
Source: mSphere. 2021 Jan 13;6(1):e01263-20. doi: 10.1128/mSphere.01263-20 (PMC7845607; doi:10.1128/mSphere.01263-20)

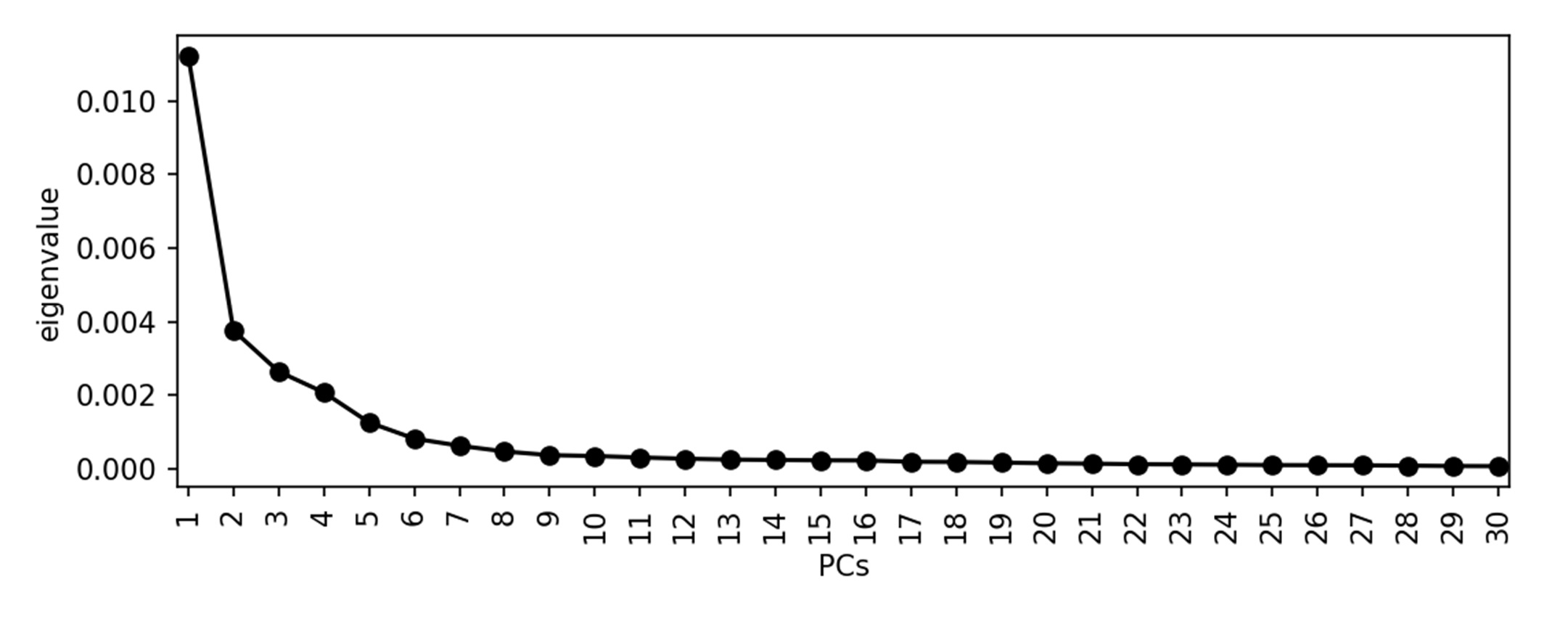

Supplement: FIG S1 [file mSphere.01263-20_sf001.tif]

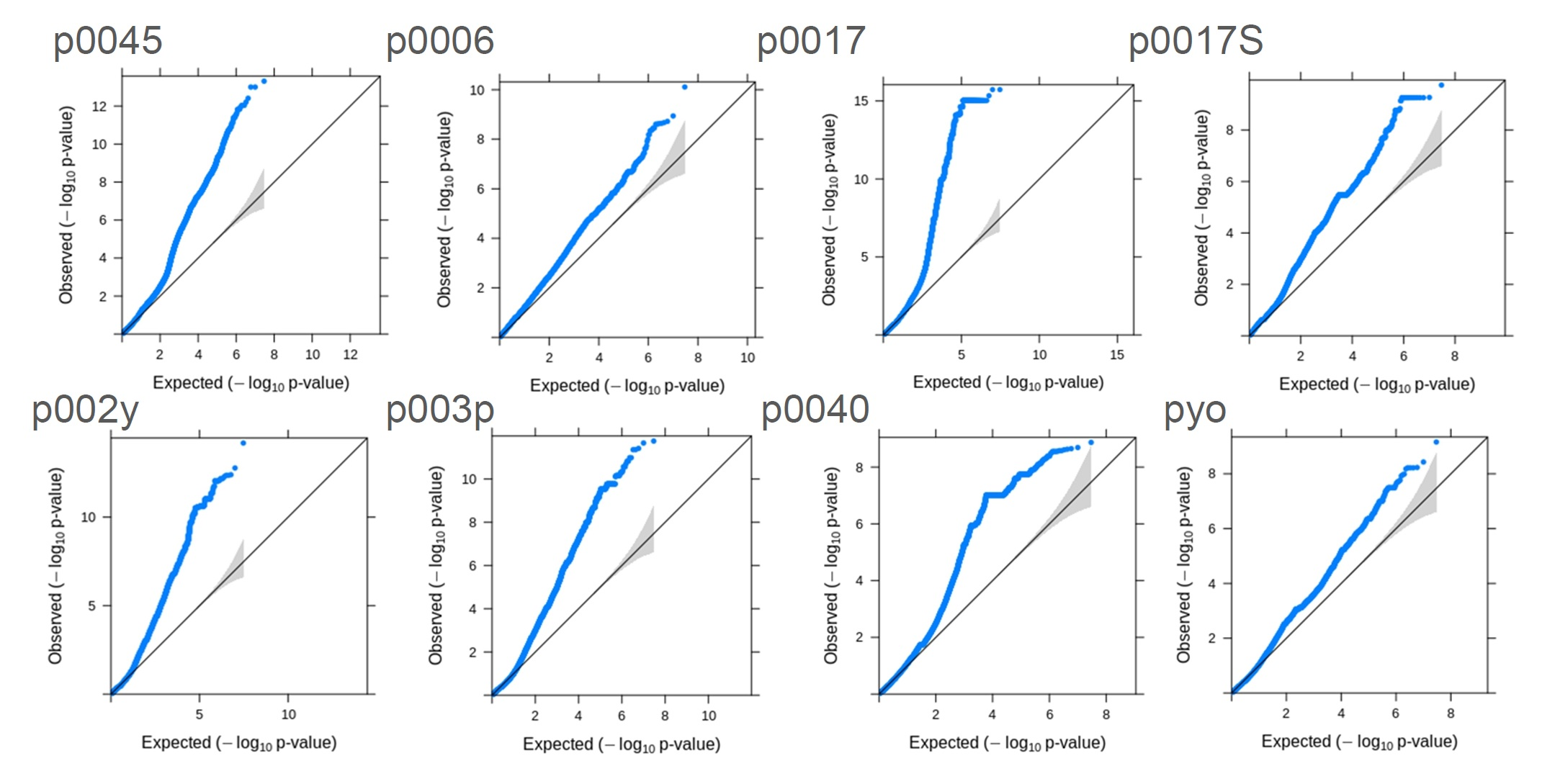

Supplement: FIG S2 [file mSphere.01263-20_sf002.tif]

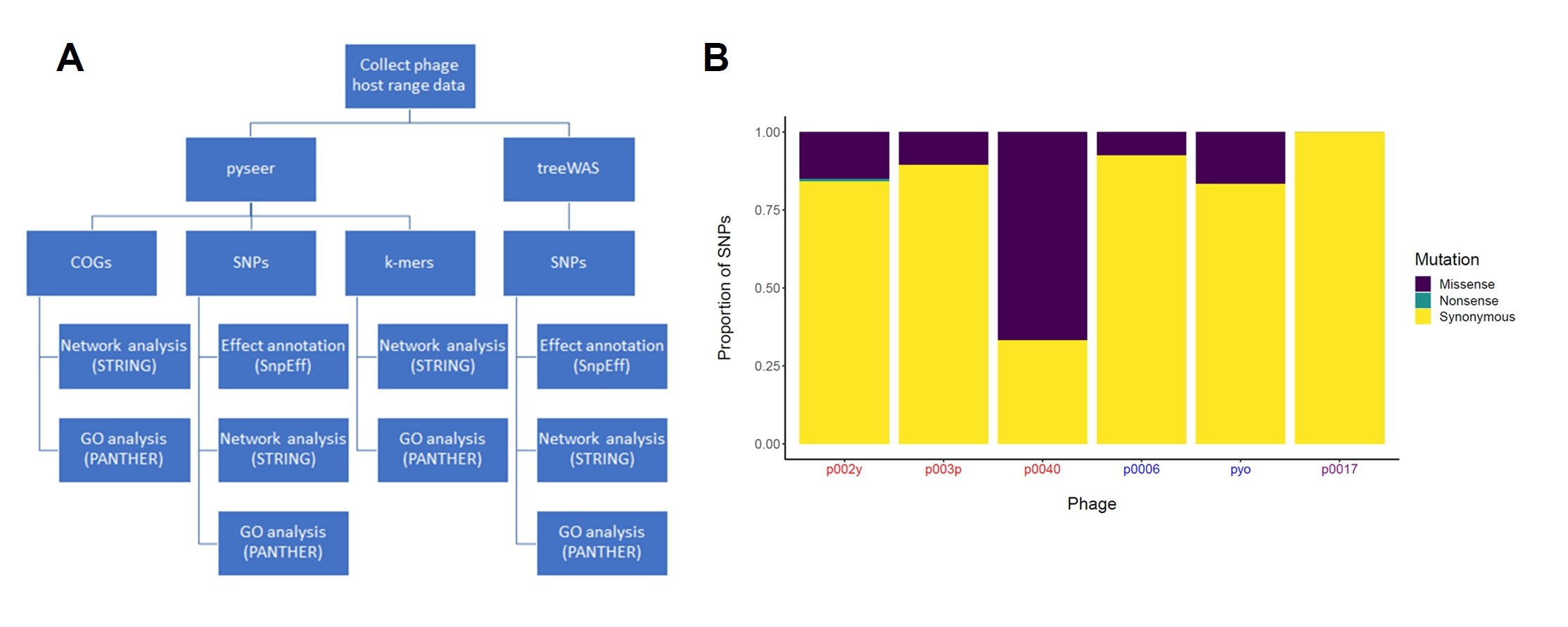

Supplement: FIG S3 [file mSphere.01263-20_sf003.tif]

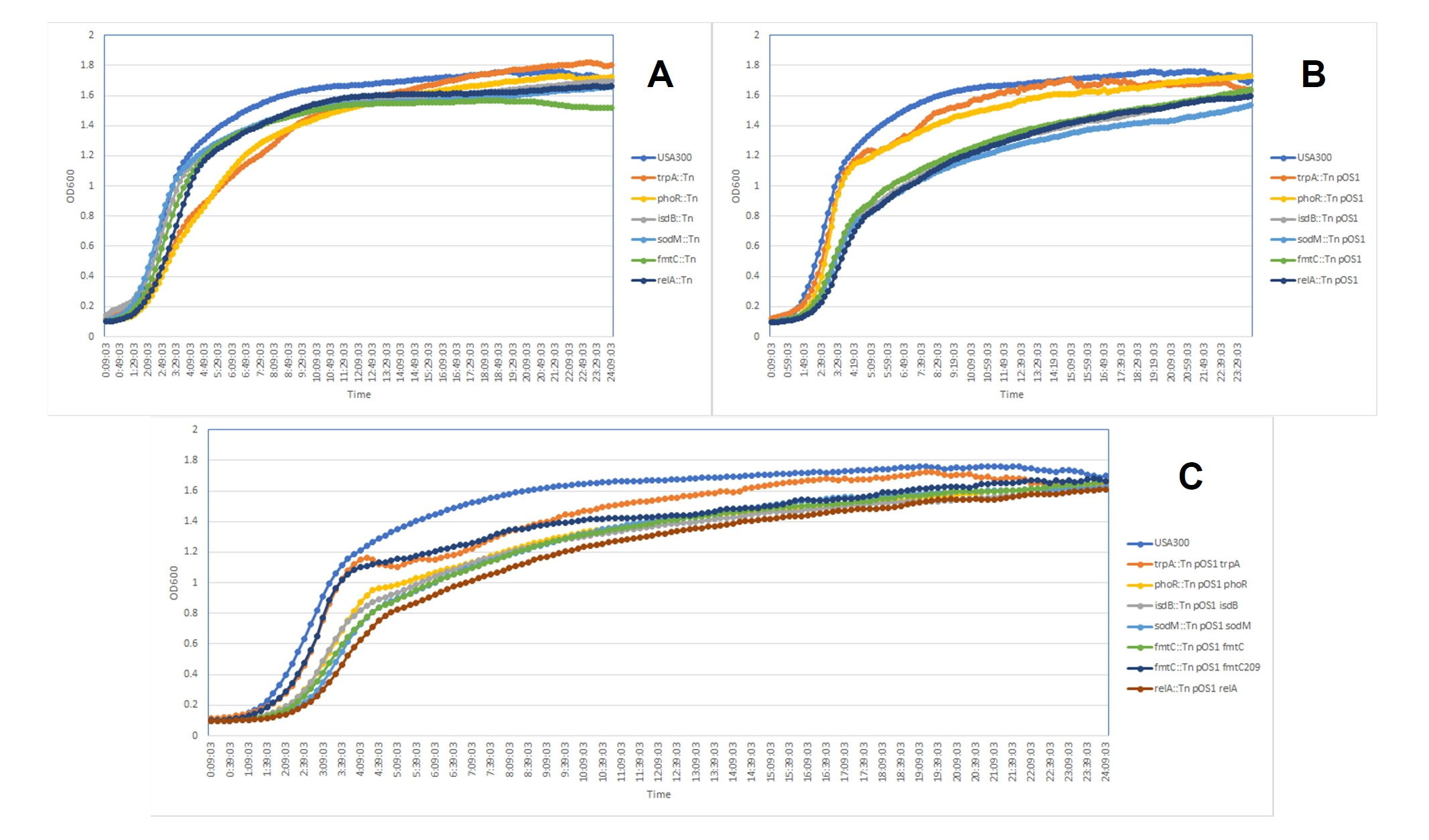

Supplement: FIG S4 [file mSphere.01263-20_sf004.tif]

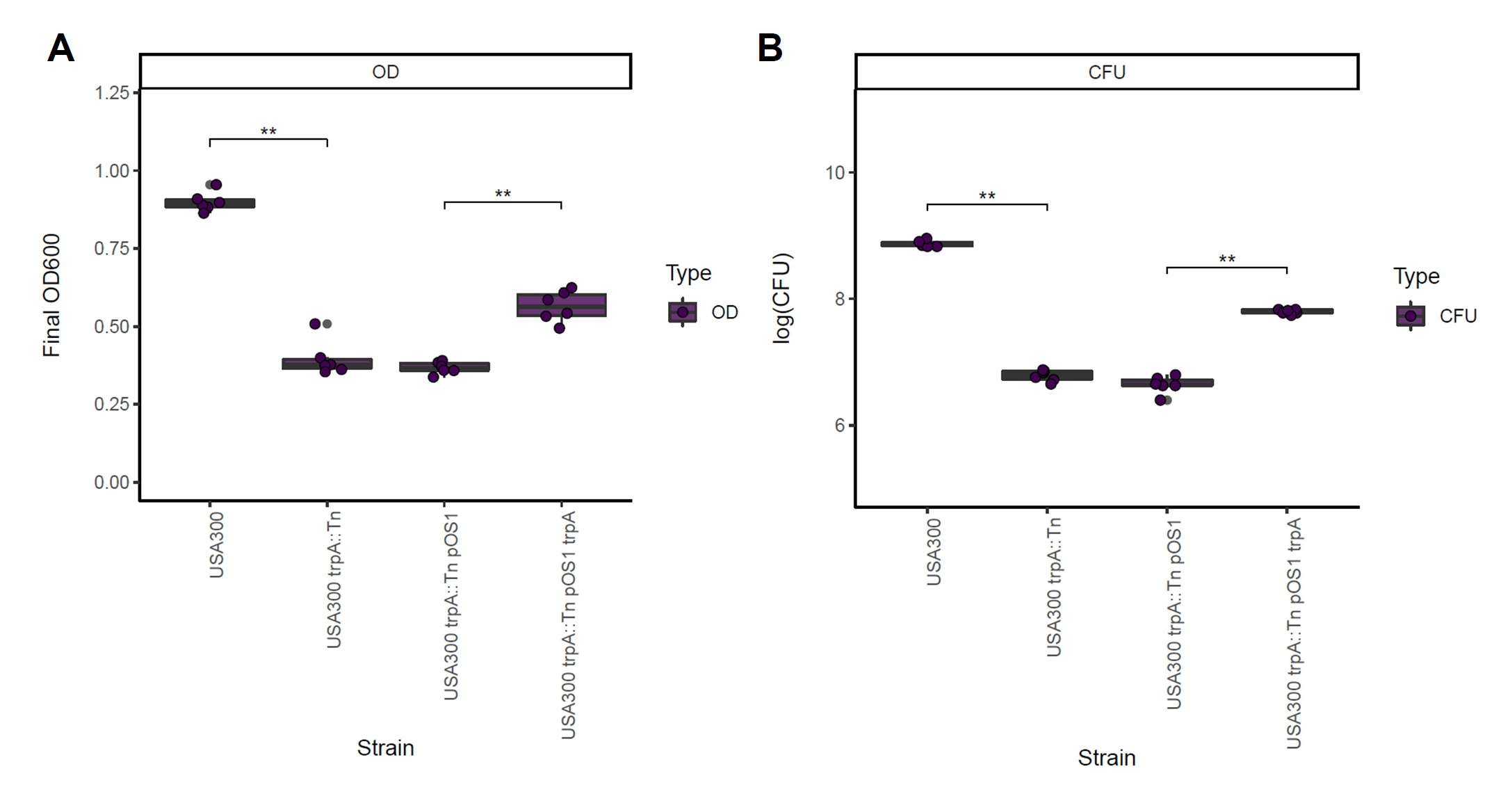

Supplement: FIG S5 [file mSphere.01263-20_sf005.tif]

Final OD600

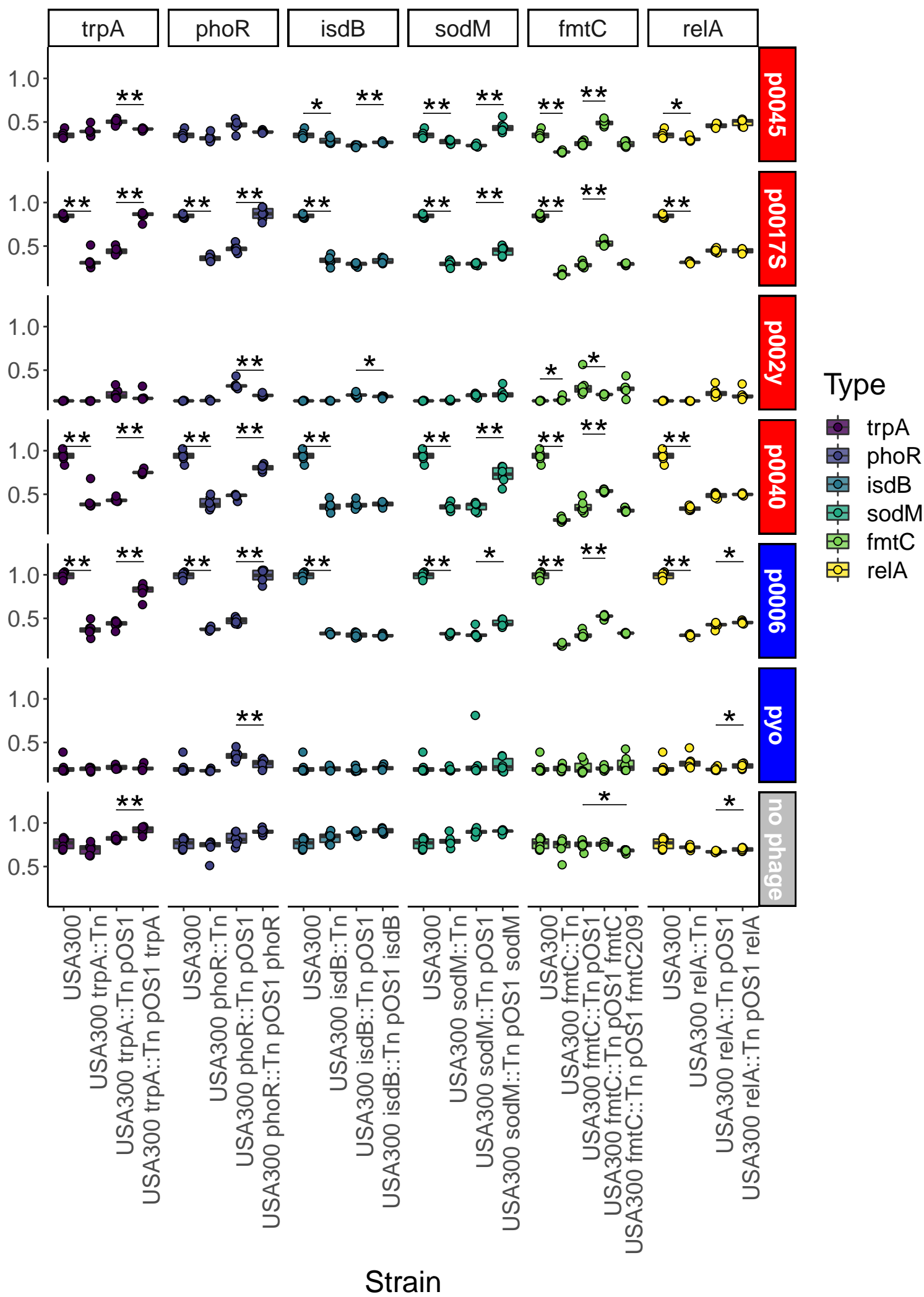

Supplement: FIG S6 [file mSphere.01263-20_sf006.pdf]

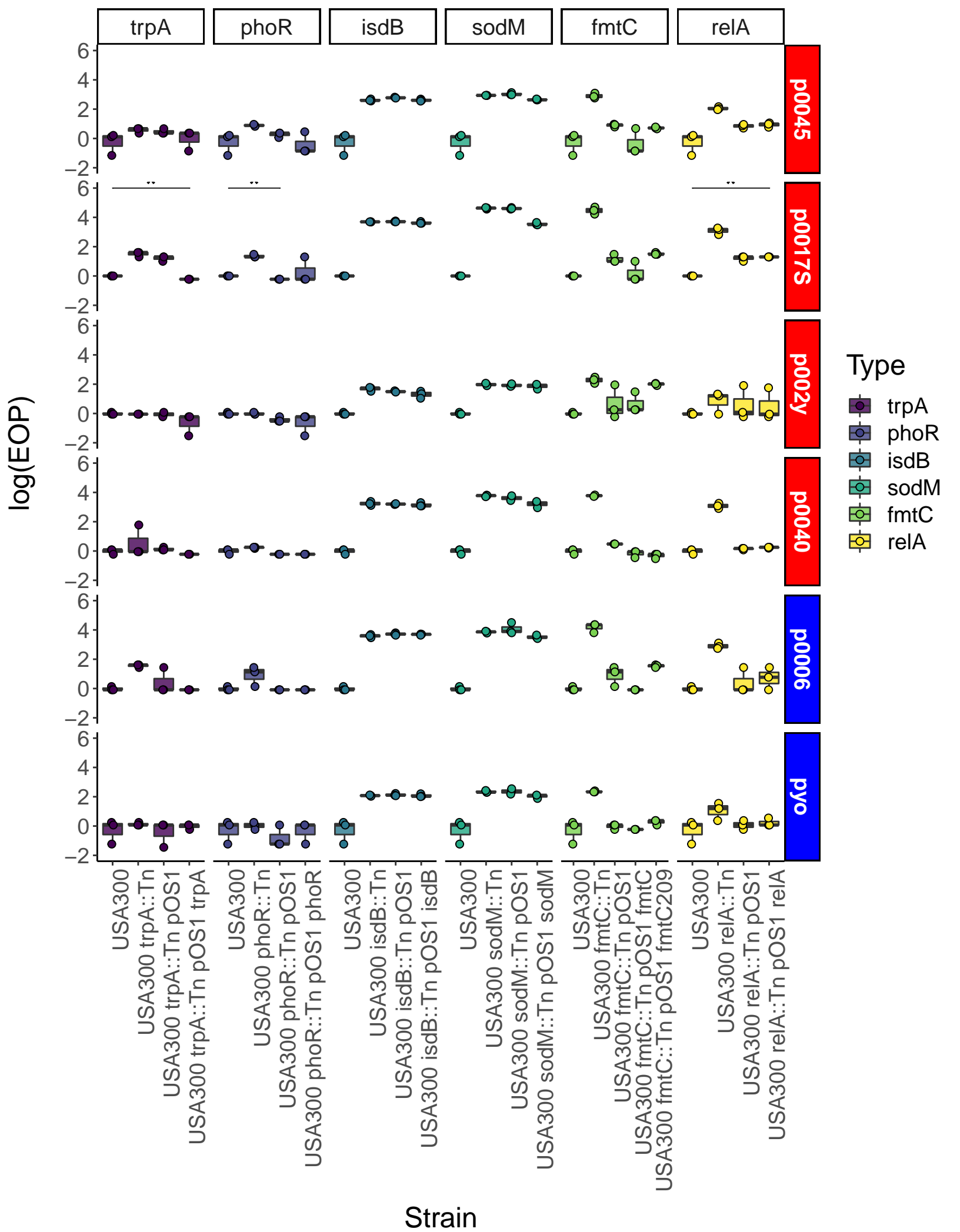

Supplement: FIG S7 [file mSphere.01263-20_sf007.pdf]

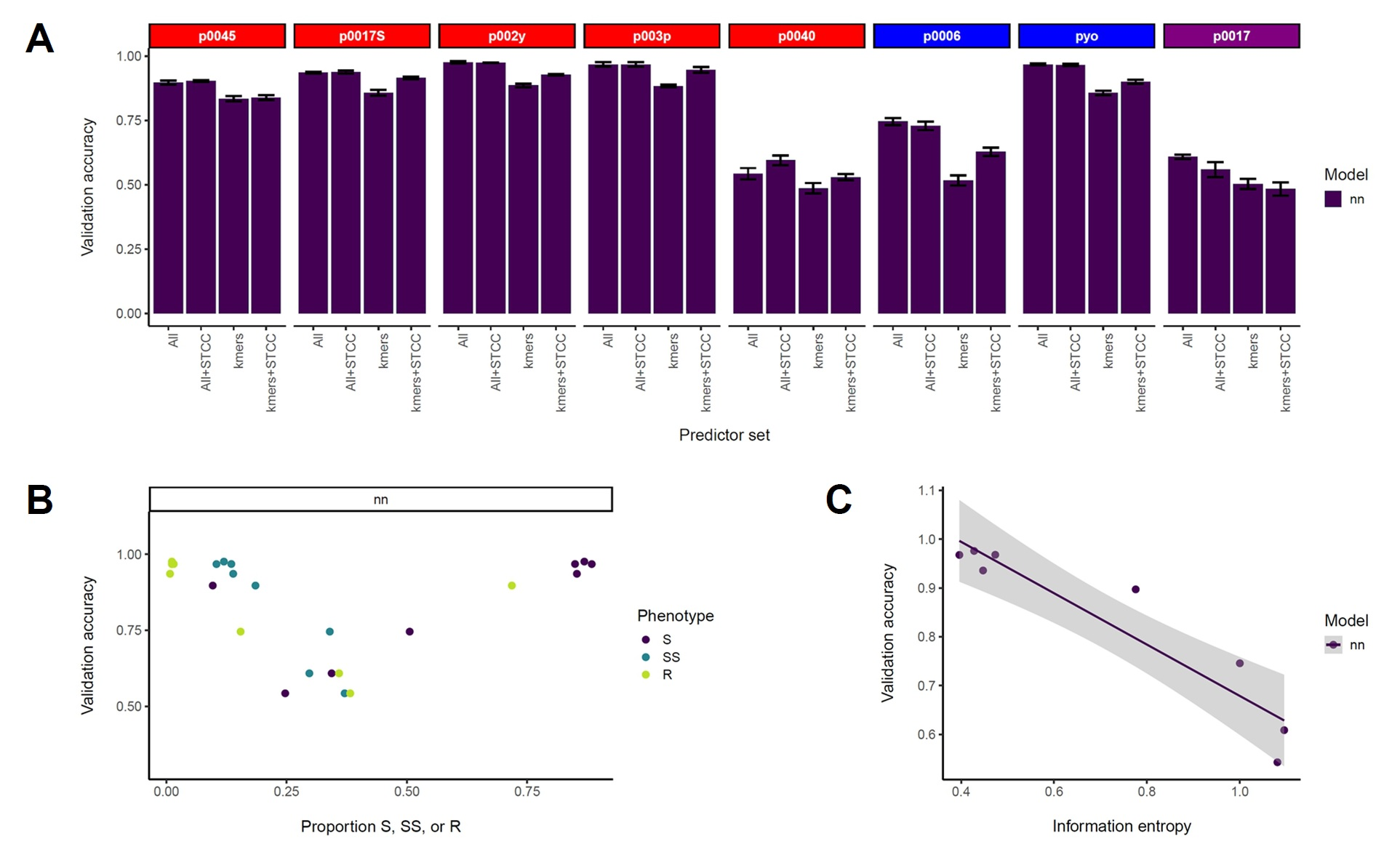

Supplement: FIG S8 [file mSphere.01263-20_sf008.tif]

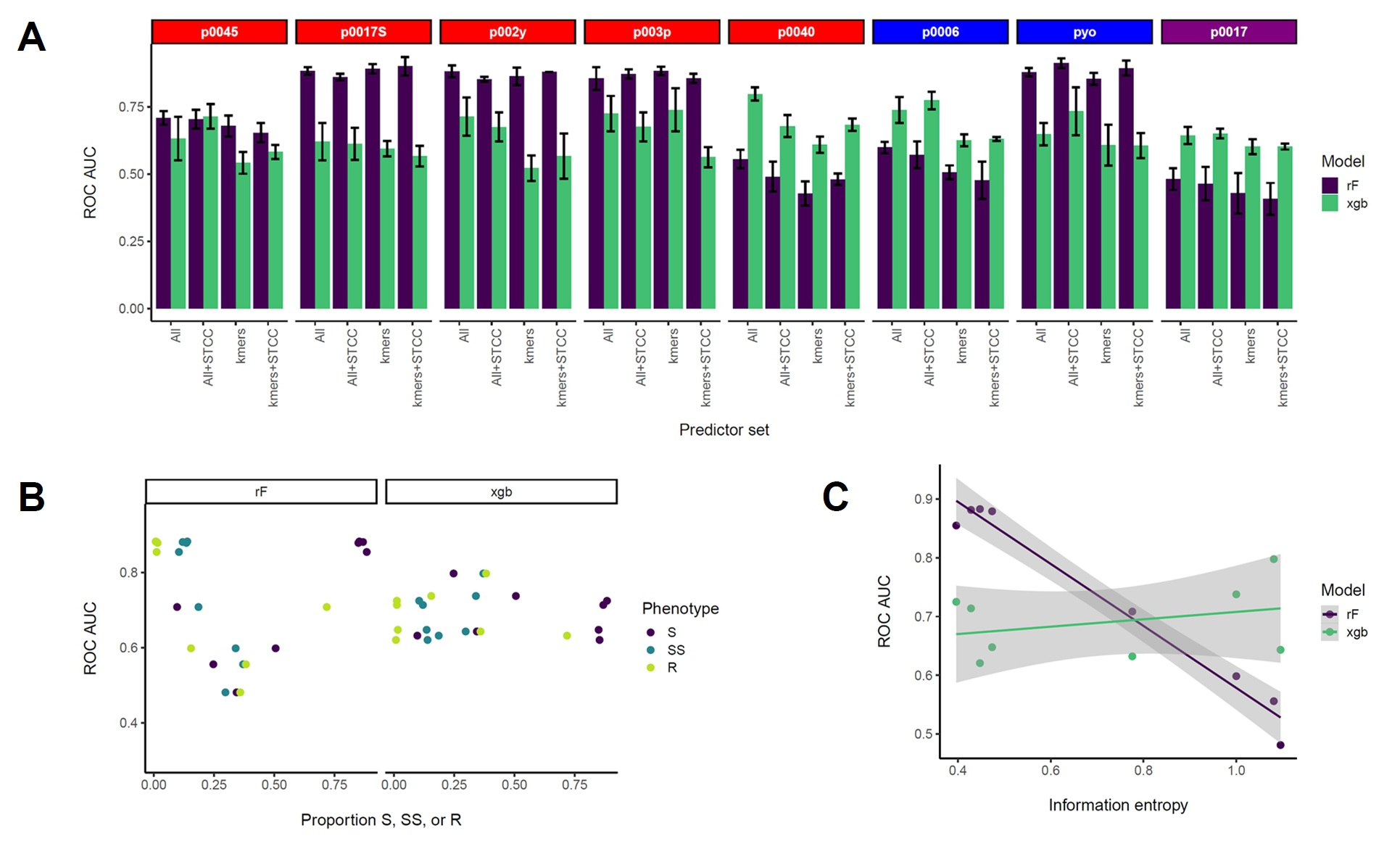

Supplement: FIG S9 [file mSphere.01263-20_sf009.tif]

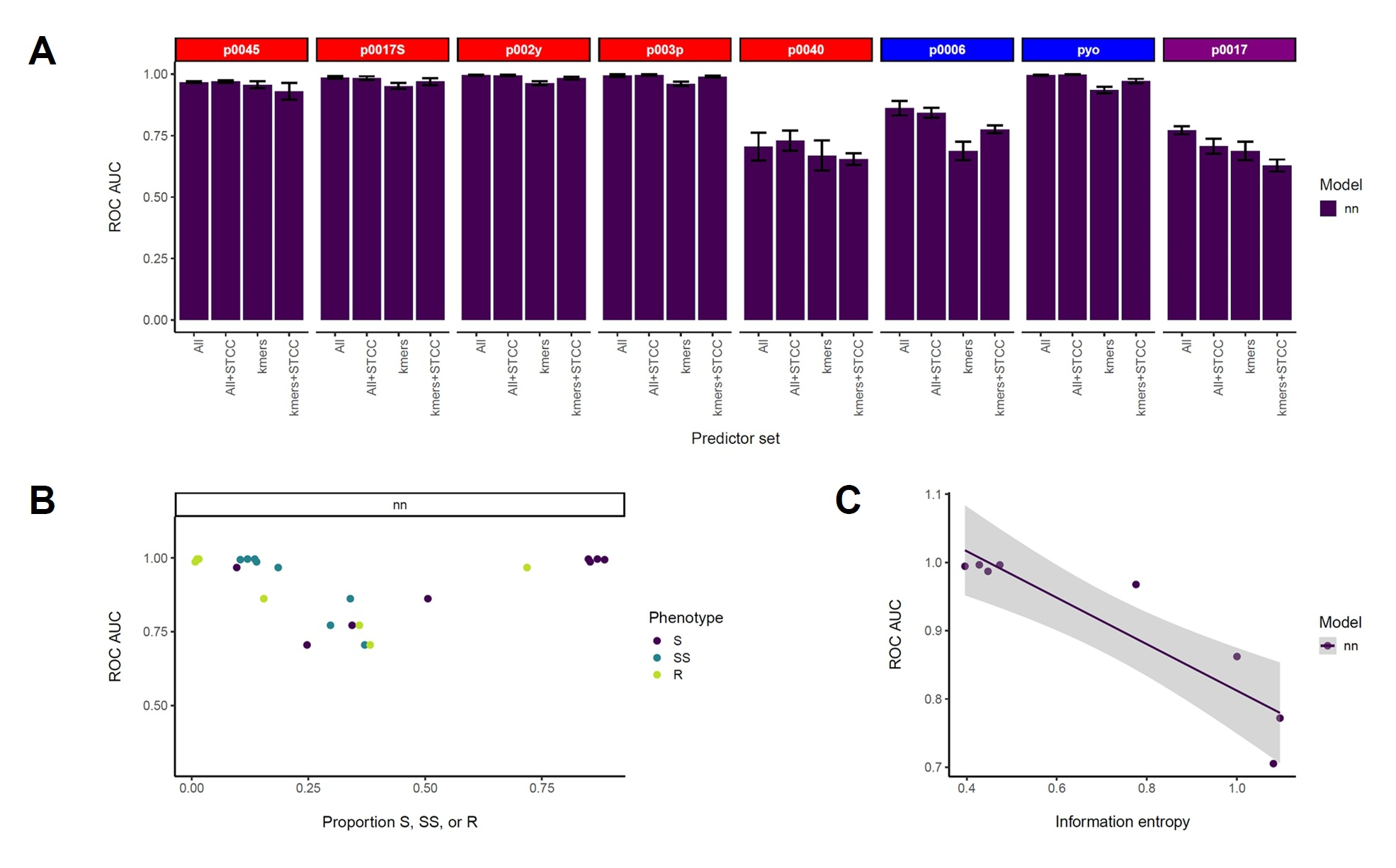

Supplement: FIG S10 [file mSphere.01263-20_sf010.tif]
